# Near-Native Protein Loop Sampling Using Nonparametric Density Estimation Accommodating Sparcity

**DOI:** 10.1371/journal.pcbi.1002234

**Published:** 2011-10-20

**Authors:** Hyun Joo, Archana G. Chavan, Ryan Day, Kristin P. Lennox, Paul Sukhanov, David B. Dahl, Marina Vannucci, Jerry Tsai

**Affiliations:** 1 Department of Chemistry, University of the Pacific, Stockton, California, United States of America; 2 Department of Statistics, Texas A&M University, College Station, Texas, United States of America; 3 Department of Statistics, Rice University, Houston, Texas, United States of America; Stanford University, United States of America

## Abstract

Unlike the core structural elements of a protein like regular secondary structure, template based modeling (TBM) has difficulty with loop regions due to their variability in sequence and structure as well as the sparse sampling from a limited number of homologous templates. We present a novel, knowledge-based method for loop sampling that leverages homologous torsion angle information to estimate a continuous joint backbone dihedral angle density at each loop position. The φ,ψ distributions are estimated via a Dirichlet process mixture of hidden Markov models (DPM-HMM). Models are quickly generated based on samples from these distributions and were enriched using an end-to-end distance filter. The performance of the DPM-HMM method was evaluated against a diverse test set in a leave-one-out approach. Candidates as low as 0.45 Å RMSD and with a worst case of 3.66 Å were produced. For the canonical loops like the immunoglobulin complementarity-determining regions (mean RMSD <2.0 Å), the DPM-HMM method performs as well or better than the best templates, demonstrating that our automated method recaptures these canonical loops without inclusion of any IgG specific terms or manual intervention. In cases with poor or few good templates (mean RMSD >7.0 Å), this sampling method produces a population of loop structures to around 3.66 Å for loops up to 17 residues. In a direct test of sampling to the Loopy algorithm, our method demonstrates the ability to sample nearer native structures for both the canonical CDRH1 and non-canonical CDRH3 loops. Lastly, in the realistic test conditions of the CASP9 experiment, successful application of DPM-HMM for 90 loops from 45 TBM targets shows the general applicability of our sampling method in loop modeling problem. These results demonstrate that our DPM-HMM produces an advantage by consistently sampling near native loop structure. The software used in this analysis is available for download at http://www.stat.tamu.edu/~dahl/software/cortorgles/.

## Author Summary

A protein's structure consists of elements of regular secondary structure connected by less regular stretches of loop segments. The irregularity of the loop structure makes loop modeling quite challenging. More accurate sampling of these loop conformations has a direct impact on protein modeling, design, function classification, as well as protein interactions. A method has been developed that extends a more comprehensive knowledge-based approach to producing models of the loop regions of protein structure. Most physical models cannot adequately sample the large conformational space, while the more discrete knowledge based libraries are conformationally limited. To address both of these problems, we introduce a novel statistical method that produces a continuous yet weighted estimation of loop conformational space from a discrete library of structures by using a Dirichlet process mixture of hidden Markov models (DPM-HMM). Applied to loop structure sampling, the results of a number of tests demonstrate that our approach quickly generates large numbers of candidates with near native loop conformations. Most significantly, in the cases where the template sampling is sparse and/or far from native conformations, the DPM-HMM method samples close to the native space and produces a population of accurate loop structures.

## Introduction

Starting from a known structural homolog, template based modeling (TBM) of protein structure provides the most accurate predictions of protein sequences with unknown structure [1], [2]. However, even with close structural homologs, structurally variable regions (SVRs), commonly referred to as loops, are the worst predicted segments [3], [4], [5]. Because loop regions join elements of regular secondary structures and often play an important role in active site composition, ligand binding, and protein-protein interactions, accurate sampling is integral to a useful TBM prediction of protein structure. Structurally, loops often lie on the solvent-exposed surface of proteins, allowing them more conformational flexibility and susceptibility to insertions and deletions. This variability makes loop regions notoriously difficult to align at both the sequence and structural level, which often results in large stretches of gapped positions. As an added level of complexity, the conformational space is usually poorly populated due to the low structural homologs. This variability and sparsity of data pose much of the challenge in modeling with current approaches, and these problems increase with loop length.

Typically, loop-modeling methods have adopted one of two general strategies, *de novo* and knowledge-based loop modeling methods. In *de novo* loop modeling [4], 6, physico-chemical based principles are used to compute the lowest energy conformations for a loop [7], [8]. In successful applications to short loop modeling, *de novo* methods include molecular dynamics simulations [9], simulated annealing [4], buildup from discretized φ,ψ pairs [10], [11], [12], and ‘random tweak’ [8], [13]. However, these methods are limited because they require significant computational resources to sample near-native conformations. Alternatively, the loops in some proteins can be classified into structural families or canonical types, as in the antibody hypervariable regions (complementarity determining regions or CDRs) [14], [15], [16], [17], [18]. Such knowledge-based schemes utilize known structures or fragments of structures to efficiently sample loop conformations, [19], [20], [21], [22], [23], but are limited to sampling within the knowledge base. Using large databases of supersecondary structures [24], loops are successively aligned with templates based on parameters such as the stem region geometry, length, and sequence similarity [25], [26], [27]. While the strategies in various methods differ in many respects, the fundamental idea is to efficiently sample the available conformational space for loops of the particular length, and then score the samples using various energy functions [7]. The modeling of longer loops up to 13 residues in length has been achieved using exhaustive sampling of φ,ψ space with clustering and energy minimization [28]. In addition, there are approaches that combine the use of loop databases and physical-based algorithms [29], [30], [31] as well as methods sampling loop libraries that focus on loop closure [32], [33], [34], [35]. For all methods leveraging information from known structures, sampling is limited to the discrete conformational space represented in the structural library. While providing efficient sampling, this approach poses difficulties in completely representing the structural variability of a loop region.

**Figure 1:**
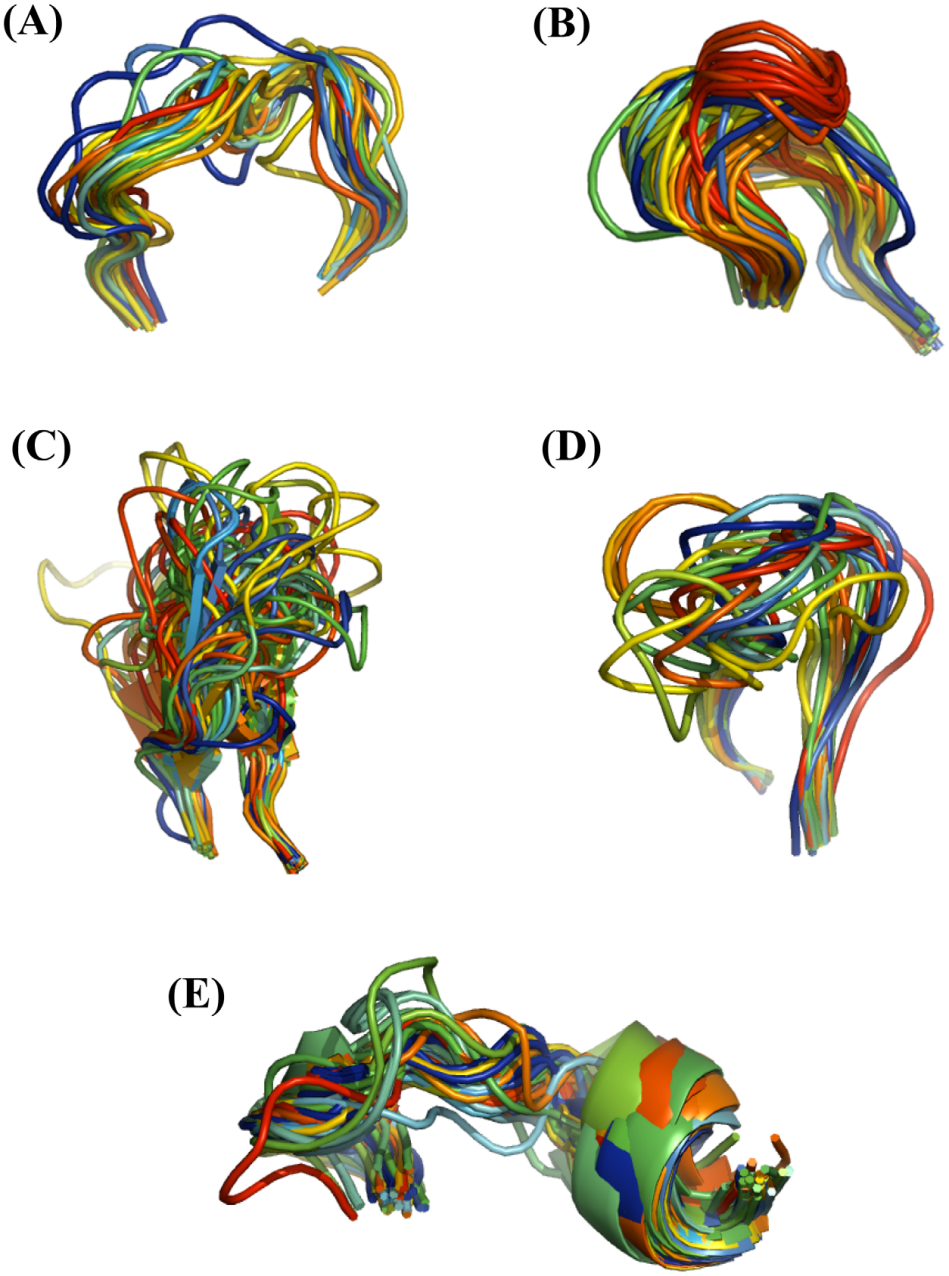
The 465 loop data set. Global superposition data set of 465 loops used to test sampling. All representations are in backbone cartoon. (**a**) 111 target loops from CDRH1 (12 residues), (**b**) 130 target loops from CDRH2 (7, 8 and 10 residues), (**c**) 111 target loops from CDRH3 (8, 10–17 residues), (**d**) 21 loops from CASP9 target, T0617 (12 residues), and (**e**) 92 globin EF loops (12, 13 and 15 residues).

To address these obstacles in sampling of loop models, a novel statistical method has been developed that implements a Dirichlet process mixture of hidden Markov models (DPM-HMM) [36], [37] for continuous density estimation of φ,ψ residue torsion angles in the loop region. This statistical modeling not only retains the advantages of utilizing information from homologous proteins but also provides the continuous sampling of conformational space allowed by physico-chemical methods. From the sparse sampling at each loop position, the DPM-HMM method computes a joint φ,ψ density using statistical inferences from neighboring residues to make probable estimations of a continuous probability. The approach uses the φ,ψ data from homologous loops to model the joint φ,ψ distributions at each loop alignment position. The results are continuous density estimations of each residue's Ramachandran space, which allows sampling from a wider range of φ,ψ values than the discrete possibilities using a loop library [7], [10], [12], [28], yet the distribution is informed by the homologous loops. A related statistical method with a different formulation called DBN-torus has been concurrently developed by Boomsma *et. al.* [34] for the modeling of fragments in template-free protein structure prediction. Unlike the specificity of this method for fragment generation, our approach is tailored for TBM and produces nearer native loop samples even when good templates are not available. Moreover, the DPM-HMM method allows fast, knowledge-based sampling of backbone torsion angles focused within probable regions of φ,ψ space [36], [37]. In this study, the ability of the DPM-HMM approach to sample near-native candidates is demonstrated in the modeling of loops from the following three groups: (1) canonical and non-canonical hypervariable loops within the heavy chain complementarity-determining regions (CDRHs) of immunoglobulins, (2) the conserved EF loop from the globin fold, and (3) the loops of CASP9 targets. Examples of these are shown in Figure 1. Sampling near native loop conformations was tested in leave one out (LOO) approach and general applicability of the method is demonstrated with the results for loop modeling of TBM targets from CASP9 experiment. Also, the performance of DPM-HMM method was compared with LoopyMod by using CDRH1 and CDRH3 data sets.

## Results

In the following sections, the DPM-HMM density estimation approach is shown to sample near native loop conformations in various classes of loop prediction difficulty. To simplify our discussion, the difficulty of prediction is classified based on the global RMSD of the closest known template to the native loop structure. Loops in the canonical class have templates that are less than 2 Å to the native structure. The common classes include templates that are 2–4 Å to the native loop conformation, although these are by no means simple to predict. Difficult loops are those that have templates greater than 4 Å and in many cases contain fewer than 10 templates to model.

### φ,ψ Distributions

**Figure 2:**
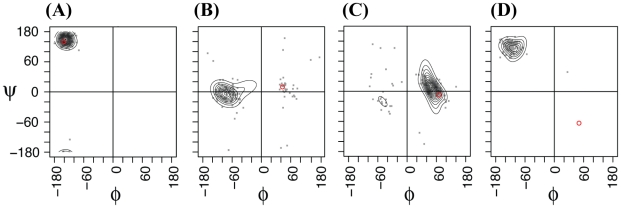
Density estimations of φ,ψ distributions. Examples of DPM-HMM estimated backbone dihedral angle density distributions at various positions of targets from predictions of the CDRH2 loop and anchor residues. The grey dots represent the observed φ,ψ input data at a particular alignment position. The contour lines represent the calculated density estimation calculated from the φ,ψ pair data. The red dots indicate the actual φ,ψ values of the target structure. Position refers to the place in the modeled loop and the PDB code refers to the predicted target. (**a**) position 1 of 1mfa [55], (**b**) position 6 of 1w72 [56], (**c**) position of 6 for 1gig [57] and (**d**) position 9 (last anchor residue) of 1rmf [58].

At the heart of our approach is the DPM-HMM density estimation of the backbone φ,ψ angles [36], [37], and the method's ability to correctly model the torsion angle space helps to explain our success or failure in modeling particular targets in our LOO tests. Figure 2 shows four examples of Ramachandran plots [38] taken from predictions of targets from the CDRH2 loops. Our method uses the normalized φ,ψ data from template loops [39] as a prior or basis for its density estimations (see Materials and Methods) of the probability distributions. As shown by the scattered points in Figure 2, the backbone φ,ψ angles from the templates provides the raw data that combine with the prior to produce the estimated distributions shown as contour lines in the plots. In a number of cases our statistical estimation of density performed well, as evidenced by the presence of the native, target φ,ψ pair (shown as a red point in Figure 2) being predicted within the highest probability regions. Panels (a) and (c) in Figure 2 show the native φ,ψ pair within the highest region of estimated density. For Figure 2a, the observed result is expected as these positions hold the anchoring residues for CDRH2, which are consistently in the β-sheet region of the Ramachandran plot. As can be seen in Figure 2c, certain positions heavily favor the left-handed helical region. This method's success in loop prediction corresponds well with density plots that contain a majority of residues with highest density around the native φ,ψ pair. By contrast, panels (b) and (d) in Figure 2 show instances in which the native φ,ψ resides in a lower probability region of our density estimates. Figure 2b shows a residue sampling the second highest region of a left-handed helix. In Figure 2d, unlike the majority residues that populate the β-sheet region, the glycine residue at the anchor position after the CDRH2 loop exhibits φ,ψ values in the commonly disallowed lower right quadrant of the Ramachandran plot. Because our density estimation model does not exclude but places a lower probability distribution in this region, these positions in the loop are more of a challenge to our sampling and helps to explain the prediction limit of 3.66 Å for poor/sparse input data described below.

### Global RMSD Comparisons

**Figure 3:**
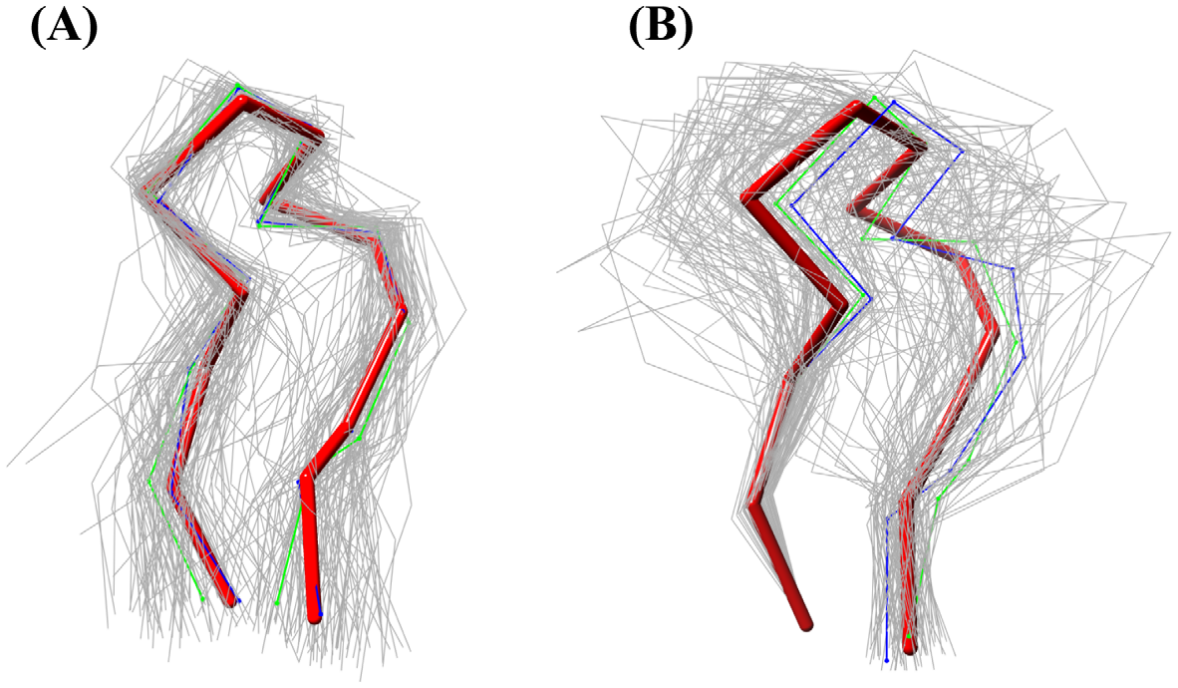
Local versus global superposition. The 97 candidate loops below 1 Å average Cα–Cα termini distance cutoff for the target loop 3bpx from dataset T0617, showing various orientations of the candidate loops (grey) in backbone Cα trace. Reference loop is shown in red stick representation. The best candidate by local superposition in blue and best candidate by global superposition is shown as green. (**a**) Local superposition of candidate loops to the reference crystal structure with average local RMSD of 1.86 Å. (**b**) Candidate loops are superposed only at the take-off region (first residue at N-terminus) of the loop. Average global RMSD of candidates to the reference crystal structure is 3.17 Å.

Instead of a measure based just on Cα atoms, root mean squared deviations (RMSDs) were calculated using all of the main-chain heavy atoms between the candidates and the native target to analyze the data and measure the accuracy of the prediction (see Materials and Methods). In addition, we performed global superposition of the loop fragments on the protein structure to calculate the RMSD between the models and the reference structure, which is a departure from the more commonly used local superposition that is independent of the overall protein structure. A local superposition of the loop candidates certainly produces lower average RMSD values to the reference structure as loop fragments often fit well locally to the reference, but the loop might not be the best candidate due to lever arm effects in the take off and landing residues. In contrast, while a global superposition will always yield a higher value for RMSD than local superposition as pointed out by Choi *et. al.* [40] assessing loop accuracy in the global context of the protein structure properly reproduces modeling conditions, where the native loop or overall structure is not known and loops are placed onto a backbone template. Figure 3 demonstrates the accuracy of global over local alignment in providing a more realistic measure to evaluate loop modeling. In both parts of Figure 3, the same 97 candidates of a loop for CASP9 target T0617, whose average Cα distance for C-terminal anchor is below 1.0 Å, are either locally (Figure 3a) or globally (Figure 3b) superposed to the native target crystal structure depicted by a thicker red backbone trace. Comparing Figures 3a with 3b, local superposition of the 97 candidates produces a much smaller spread over a global superposition. The average RMSD proves this observation: 1.86 Å for local superposition opposed to 3.17 Å for global superposition. However, the most significant difference occurs at the take-off and landing positions. In the local superposition, the variation around the ends is larger, whereas in the global superposition, it is quite small. Comparing the closest candidates by both methods demonstrates the importance of using global superposition. The blue line is the best candidate by local superposition, which has a local RMSD of 0.58 Å yet a global RMSD of 1.10 Å. So, while this candidate looks to be the best match in Figure 3a, this loop would not be the best fit on the protein structure as shown in Figure 3b. It can be seen that even though the first N-terminal residue (anchoring residue) coordinates are shared between the target and all candidates, the overall orientations of the loops are very diverse. The green backbone is the best overall loop candidate found by global superposition at a RMSD of 0.77 Å (Figure 3b), which is closer to the red native backbone than the top loop selected by local superposition. This candidate would have been missed in a local superposition with a RMSD of 0.65 Å. By not considering the fit of the loop onto the structure, local superposition accuracy is misleading and impractical in TBM as loops need to be evaluated in the context of a complete structure. Therefore, even though the RMSD values are higher for global superpositioning, the comparison stays truer to real prediction situations where the loop is being matched onto the body of model structure.

### Sampling Efficiency

**Figure 4:**
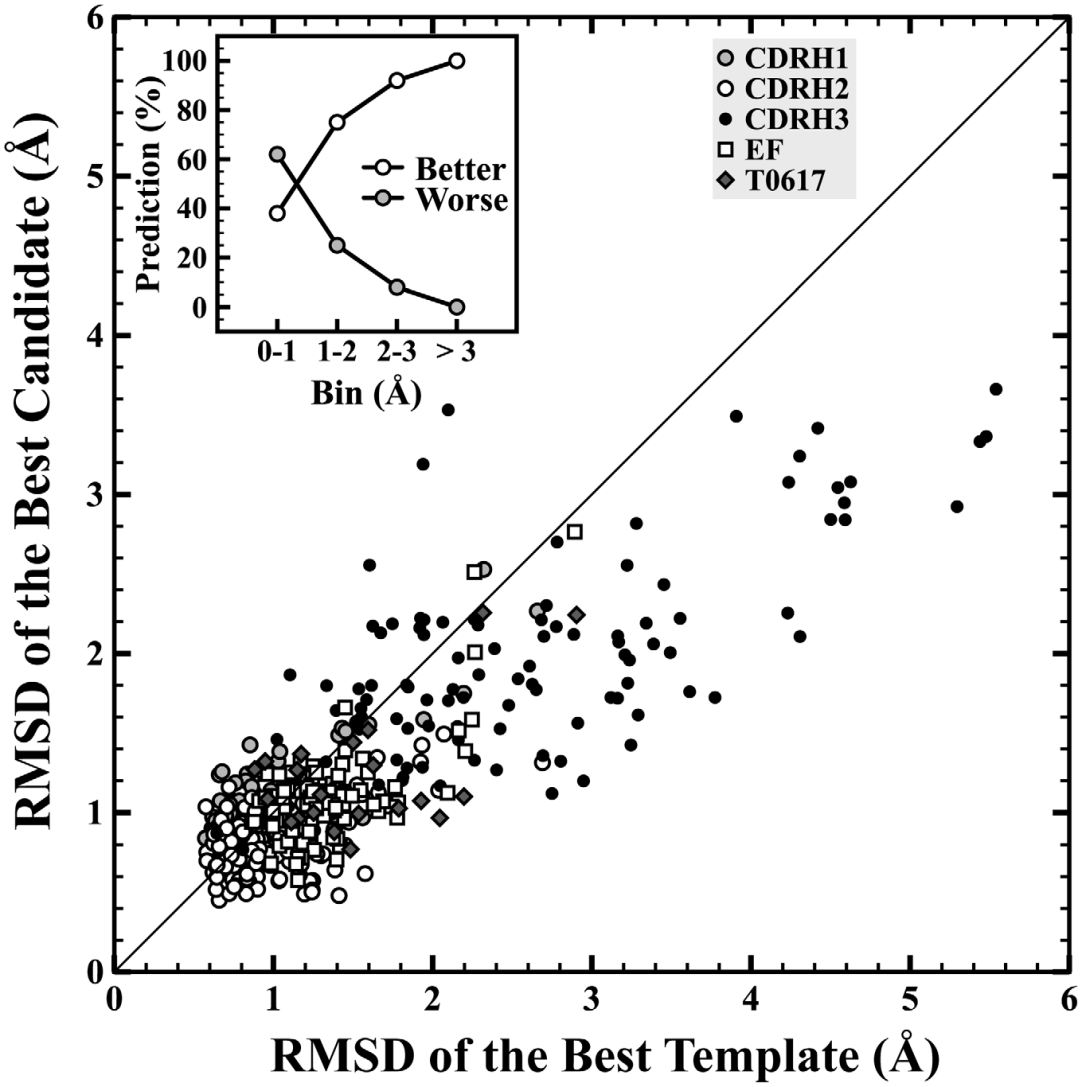
DPM-HMM Sampling performance. RMSD of the best candidate versus RMSD of the best template. The diagonal line is unity. Points below the line indicate predictions better than the best template. The inset shows the percentage of better and worse predictions in each RMSD bin. When the RMSD of the best templates are below 1 Å, the chances our methods improve the loop are about 38%. When they are between in 1–2 Å, the chances are higher than 75%. In the 2–3 Å range, chances of improvement are higher than 93%. For higher than 3 Å, the loop structures are always improved.

The DPM-HMM method is able to produce consistent results across the various types of loop targets. In our LOO tests modeling the 465 loop data set (Table 1), the low mean global RMSDs for the best candidates shown in Table 1 demonstrate that our method performs well at sampling near-native loop candidates. To provide more details about the DPM-HMM's performance, sampling accuracy was measured by comparing the global RMSD of the best sampled candidate to that of the best template from the discrete set of template loop structures (Figure 4). The best template is the one with lowest global RMSD to the target native loop segment. In Figure 4, the points below the diagonal line indicate loops our method modeled better than the best available template (best candidate's RMSD is lower than that of the best template). The DPM-HMM method performs consistently well for common targets with templates averaging between 3 to 4 Å RMSD and even for the difficult targets with a mean template RMSD above 7 Å. Of all the 465 targets predicted by the DPM-HMM method, the best candidate global RMSDs are in the range from 0.45 Å to a top value of 3.66 Å, regardless of the loop length, number of templates, and the quality of the templates. So, we can reliably say that our method samples loop conformations at least within 3.66 Å to the native.

**Table 1:** Loop modeling template datasets and accuracy measure (RMSD) for the sampled candidates.

LOOP	Length (AA)	Targets	Templates' Average RMSD			Best RMSD^d^	Average Best RMSD^e^
			Min^a^	Max^b^	Average^c^		
**CDRH1**	12	111	2.35	2.44	2.42(0.01)	0.61	1.04(0.28)
**CDRH2**	7	30	1.49	1.61	1.58(0.02)	0.45	0.62(0.20)
	8	87	2.49	2.69	2.68(0.02)	0.54	0.84(0.19)
	10	13	1.97	2.88	2.75(0.24)	0.50	0.81(0.16)
**CDRH3**	8	13	2.26	3.86	3.65(0.42)	0.77	1.08(0.17)
	10	15	4.53	5.31	5.18(0.19)	1.12	1.46(0.22)
	11	13	3.14	3.54	3.41(0.11)	1.17	1.52(0.29)
	12	6	4.47	5.39	4.96(0.41)	1.81	2.10(0.17)
	13	28	4.81	5.30	5.20(0.10)	1.32	1.92(0.40)
	14	14	5.46	7.37	7.11(0.49)	1.72	2.16(0.39)
	15	8	3.93	4.50	4.31(0.20)	1.64	2.36(0.46)
	16	5	6.08	6.93	6.42(0.34)	2.92	3.15(0.19)
	17	9	7.05	7.68	7.43(0.24)	2.82	3.13(0.32)
**EF**	12	23	2.58	2.79	2.73(0.05)	0.58	0.98(0.28)
	13	66	2.58	2.97	2.95(0.05)	0.68	1.08(0.15)
	15	3	1.46	4.15	3.11(1.45)	2.01	2.43(0.39)
**T0617**	12	21	3.17	3.41	3.33(0.07)	0.77	1.23(0.39)

Of the best candidates, lowest RMSD (Å) and average RMSD (Å) for five loops sampled using DPM-HMM method along with their loop length, number of targets in each group and average RMSD (Å) of all the templates used.

a Minimum average RMSD of all the templates in a subgroup.

b Maximum average RMSD of all the templates in a subgroup.

c Average of mean RMSD of all the templates in the group. Standard deviations are given in parenthesis.

d Lowest of all best candidates' RMSD that is sampled in each subgroup of loop targets.

e Average of best candidate's RMSDs for every target in each subgroup. Standard deviations are given in parenthesis.

The inset in Figure 4 shows the percentage of better or worse candidates compared to the best template binned by RMSD. In the very close canonical RMSD range of 0–1 Å, 38% candidates were sampled better than the best templates. Moreover, in this regime very close to the native structure where there is a higher probability to produce incorrect structures over the right ones, the DPM-HMM method sampled the remaining 62% in this canonical class not far from the best template. The worst case is with maximum deviation of 0.6 Å and mean deviation of 0.2 Å from the best template. As shown in the inset to Figure 4, our sampling percentages from the DPM-HMM method only improve as the difficulty of loop modeling increases. In the RMSD range of 1–2 Å, around 75% of the best candidates improved on the best templates, and of the 25% that did not, the average increase in RMSD was 0.3 Å with a worst case of 1.3 Å deviation from the best template. In the next bin between 2–3 Å RMSD, 93% or almost all cases produced better candidates. In this range, the 7% of the cases that produced worse candidates averaged 0.6 Å RMSD with a maximum at 1.4 Å. For the cases with templates above 3.0 Å RMSD, which combines some common and all the difficult loop targets, our DPM-HMM method consistently constructs candidates that were better than the closest templates. Overall, about 76% of the loop conformations are sampled more accurately than the best templates available. This consistency of the DPM-HMM method in building improved loop models over these sets of varying difficulty demonstrates its utility and promise.

### Influence of the Template Knowledge Base

**Figure 5:**
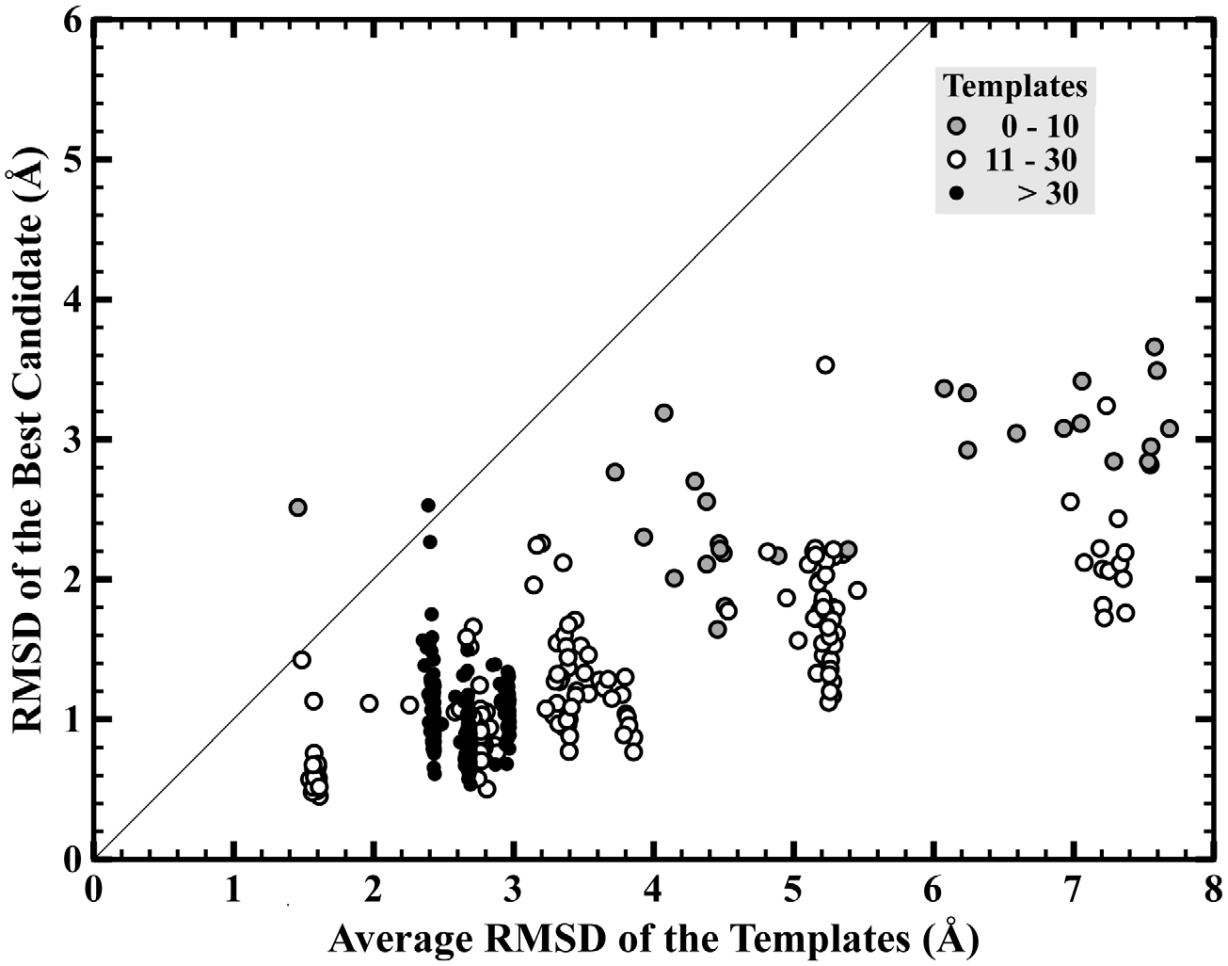
Influence of the variation of input data. RMSD of the best candidate versus average RMSD between all the templates. The data points are classified according to the number of templates used for input in the DPM-HMM φ,ψ density estimation. Grey filled circles represent targets with less than 10 templates, open circles are with 10 to 30 templates and black filled circles are with more than 30 templates.

We wanted to investigate how much influence the input data set had on our ability to build near native models. Figure 5 shows the correlation between the input templates' average RMSD and the best predictions for each of the 465 targets in our LOO tests. As a measure of the diversity of the templates, the average RMSD is calculated as the mean value between all the templates used in the DPM-HMM density estimation with each other. A larger average RMSD indicates greater diversity in the input template data. As expected, near native input data produces better model structures. As shown previously, the DPM-HMM has a limit of 3.66 Å even with very poor input data with average RMSD values past 7.0 Å. Furthermore, the targets were classified into 3 groups according to the number of templates used to produce the DPM-HMM models: (1) those relying upon less than 10 templates, (2) those with between 11–30 templates, and (3) those with greater than 30 templates. For the loops molded with fewer than 10 templates, their best RMSDs are mostly above 2 Å and do not demonstrate a strong dependence on the quality of input data. This suggests that the influence of the prior distribution determines the upper limit of our approach's abilities to sample the native structure. The targets that used between 11–30 templates display the expected correlation of improved candidate production from nearer native sets of templates. For this amount of input data, the DPM-HMM approach increases the probability of sampling near the target structure, which results in RMSDs of most of the best candidates below 2 Å. In our data set, there were only three loop examples that possessed more than 30 templates for input data: CDRH1 12 residue loop with 111 targets, CDRH2 8 residue loop with 87 targets, and EF 13 residue loop with 66 targets. Their average RMSD values are similar and cluster around 2.5 Å (see black filled circles in Figure 5). The large clustering is due to the numerous LOO tests that could be performed in this group. The best RMSD values range from 0.5 to 1.8 Å with a few exceptions discussed below, and 260 target tests in this class are modeled as below 1.8 Å RMSD. Although a large number of templates gives a better chance to model the long loops close to the native structure, the results from this class suggests that there is a saturation limit to the amount of information provided by the input data.

The DPM-HMM approach fails to produce a model that is better than the average RMSD of the templates in two special cases (data points above the unity line in Figure 5). One particular case is a 15 residue EF loop that was modeled using only 2 templates. For this target, the average RMSD of templates is 1.46 Å but the RMSD of the best candidate is 2.77 Å, which is shown as the grey filled circle above the unity line in Figure 5. This high RMSD arises primarily from using idealized bond lengths and bond angles to build loop structures from φ,ψ angles that are unable to reproduce that native loops conformation due to irregularities in bond angles, which has been previously discussed in detail [41]. The other loop that was poorly modeled belongs to the CDRH1 segment from the humanized anti-gamma-interferon antibody (1b2w [42]) in the class of greater than 30 templates. The best sampled model has a global RMSD of 2.53 Å to the native loop structure (black dot above the diagonal in Figure 5). This loop possesses a 3_10_ helical conformation in the middle of the CDRH1, which places it as a distinct outlier in the dataset with over 100 canonical templates.

### Sampling Efficiency Dependence on Loop Length and Template Number

**Figure 6:**
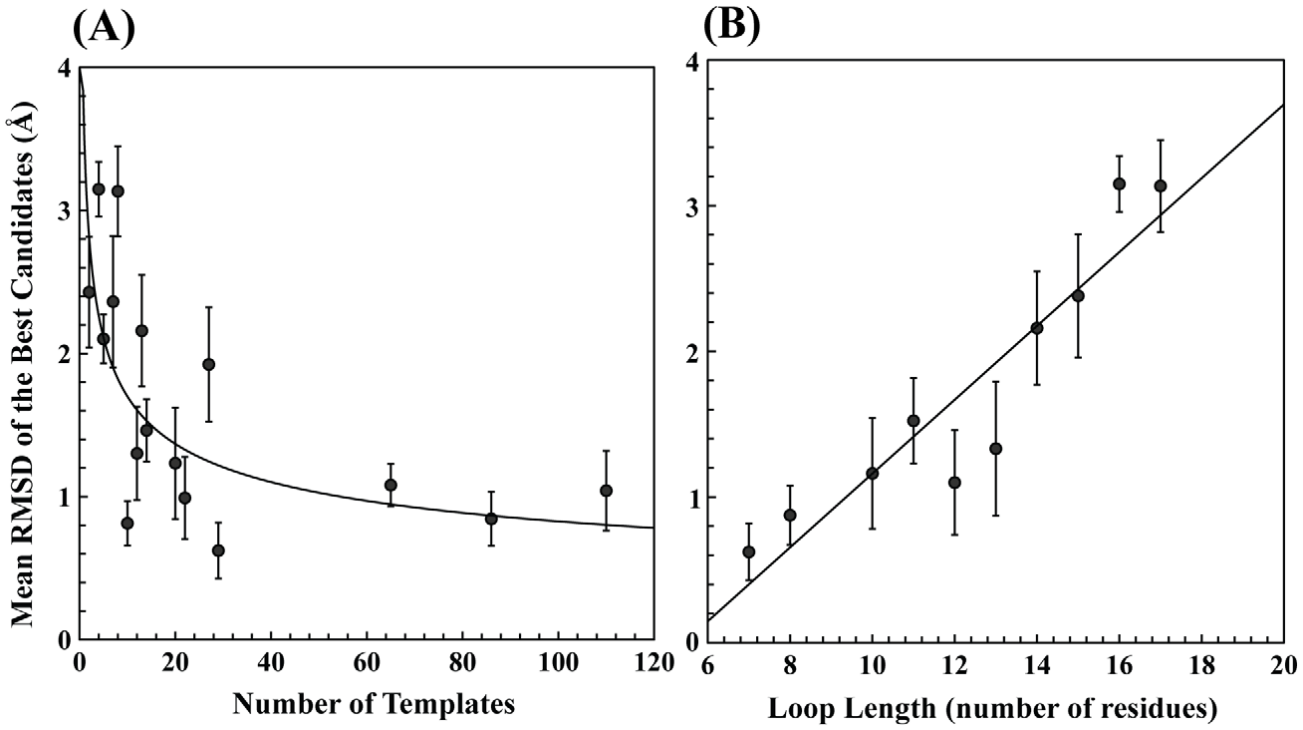
Dependence of input data: length and amount. (**a**) Correlation of the best candidate RMSD with loop length. The prediction shows a linear correlation to loop length. (**b**) Correlation of RMSD of the best candidate to the number of templates. The candidates decrease in RMSD as the number of templates increases to a cutoff of ∼30 templates, suggesting that more than 30 templates do not improve the sampling in the DPM-HMM method.

The relationship between the loop length and the sampling efficiency was also investigated. In general, loop-modeling methods are more effective at predicting the shorter loops, where the accuracy decreases as the loop length increases. Figure 6 shows sample of various sizes of loops ranging from 7 to 17 amino acid residues. A linear correlation exists between the loop length and best-sampled loop conformation (Figure 6a). In loop modeling, loops with 11–13 amino acid residues are considered long and prediction accuracies of about 1.0–1.5 Å for these long loops are considered to be a success [40]. In this study, sampling efficiency for shorter loops (7–10 amino acid residues) was found to be below 0.5 Å. For the loops with 11–13 residues, the best candidates' global RMSDs are below 1.2 Å. For longer loops with 14–17 amino acids in length, the global RMSD is within the range of 1.8–3.0 Å, which improves upon the sampling reported by other methods [40], [43]. The upper bound of sampling efficiency achieved here is about 3.66 Å, which encompasses the largest global RMSD for one of the predicted candidates belonging to the longest (17 residue long) of CDRH3 loop category. The best candidates' RMSDs are also plotted against the number of templates in Figure 6b. As expected, the higher number of templates improves upon the sampling. From Figure 6b, the DPM-HMM method requires at least 30 templates in a data set to consistently make a prediction below 1 Å. With less than 30 templates, the dependency is more about how close the input data is to the target loop structure, where some instances are successful and others approach the 3.66 Å limit of our method.

**Figure 7:**
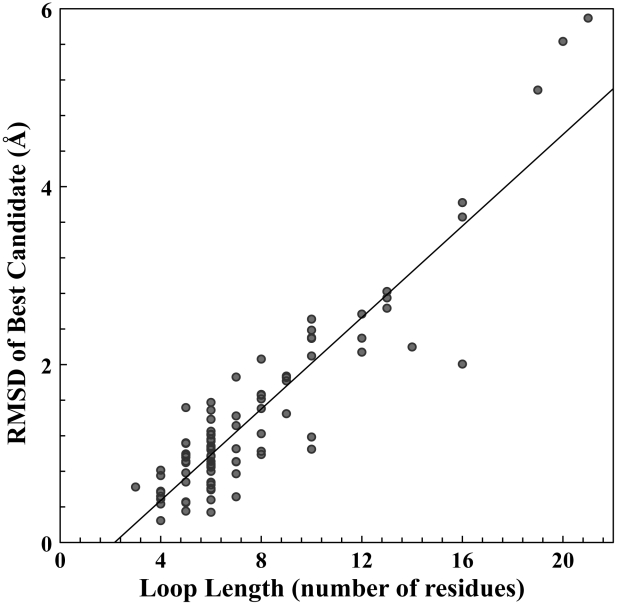
CASP9 Loop sampling. Assessment of sampling efficiency for the 90 loops modeled in the CASP9 experiment (see Materials and Methods for selection). All loops were modeled with very limited number of templates, mostly 1–5, and with templates of various lengths. For smaller loops with 3–8 residues, global RMSD is mostly below 2.5 Å. For medium sized loops (8–13 amino acids), global RMSD is between 1–3 Å. As the loop length increases, best-sampled conformations have higher RMSD from the native structure. The DPM-HMM fails after 20 residues as shown by the increase in RMSD above 5 Å.

To further investigate the DPM-HMM method, sampling was analyzed for 90 properly identified loops modeled in 45 TBM targets during our group's CASP9 campaign. Figure 7 shows RMSD of the best candidate as a function of loop length. This dataset represents realistic modeling under real-world conditions where very few templates are available to model the torsion angle space. Also, available templates were of various sizes in loop length for each target loop. Our results demonstrate that sampling efficiency is very good for the loops of smaller lengths (3–7 residues) with best-sampled candidates global RMSD of 0.25 Å to the native reference structure. Average global RMSD for best-sampled candidates in this group is about 0.89 Å. For medium sized loops (8–13 residues), best candidate RMSD is 0.99 Å and the mean over the group is 1.9 Å. For longer loops with more than 16 residues, sampling efficiency escapes the DPM-HMM limit of 3.66 Å. These longer loops pose a problem to the DPM-HMM to accurately model the data over so many residues. As can be seen from Figure 7, the limit is stretched at 20 residues, where the best candidates are greater than 5 Å. Overall, the results demonstrate the general applicability of our method in realistic TBM situation where limited number of templates with variable loop lengths was used for modeling.

### Sampling Efficiency Compared to LoopyMod

**Figure 8:**
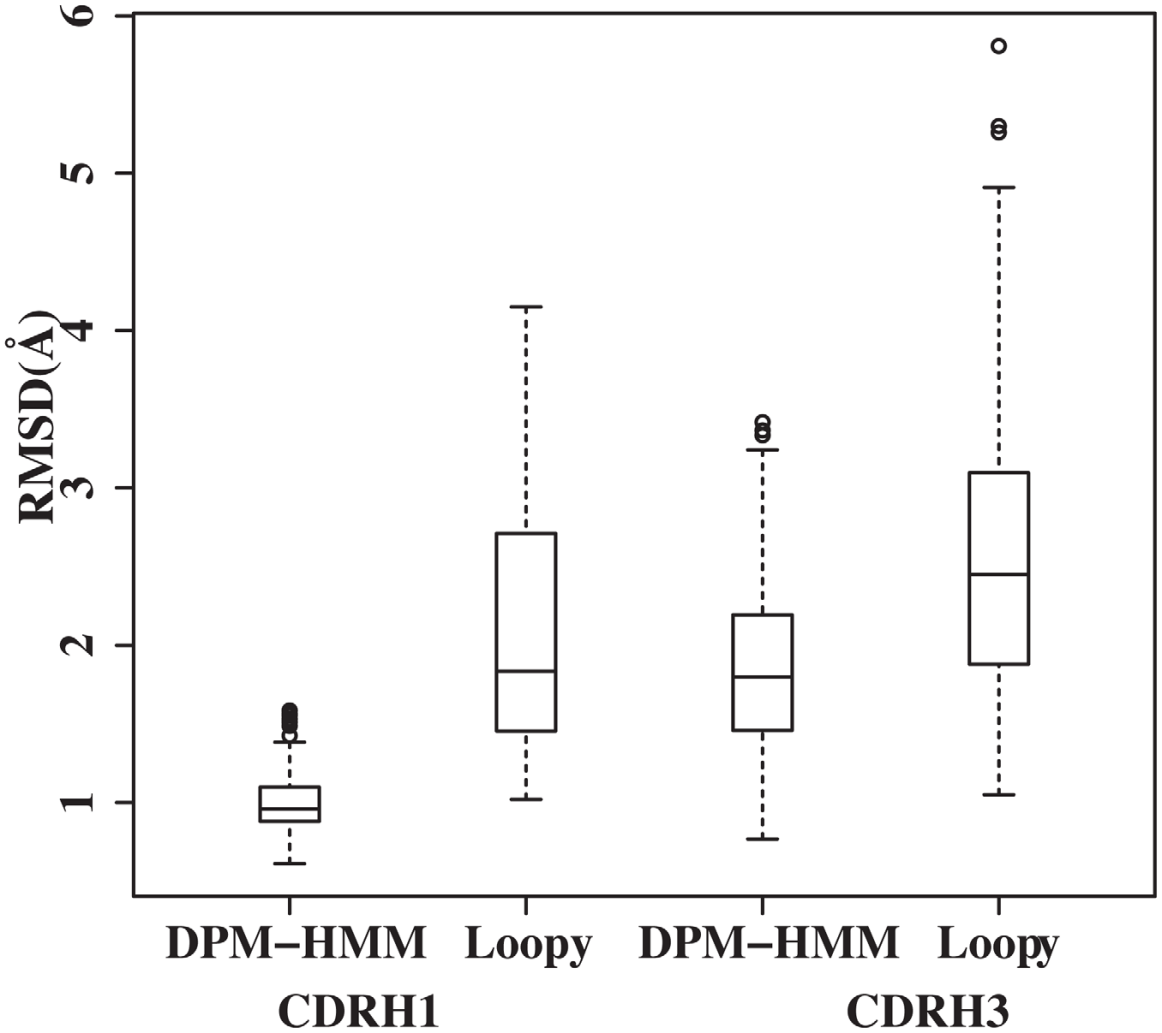
Loop sampling comparison. Boxplots display the RMSD sampling distribution of the DPM-HMM method alongside that of the LoopyMod method for loops of different difficulty: canonical (CDRH1) and non-canonical (CDRH3) loops. Comparison of sampling to the canonical CDRH1 is shown by the left 2 boxplots and the comparison to the non-canonical CDRH3 by the right 2 boxplots. In both cases, the DPM-HMM exhibits a tighter distribution and lower median RMSD.

Figure 8 compares the sampling efficiencies of DPM-HMM and LoopyMod for 2 sets of loops: the canonical CDRH1 and the non-canonical CDRH3. First, canonical conformations from CDRH1 dataset containing 111 target loops were sampled with LoopyMod and results compared to the DPM-HMM method in the first 2 columns of Figure 8. The global RMSD of the best candidate by LoopyMod is 1.02 Å, which is higher than the 0.61 Å by DPM-HMM method. Variance within RMSDs of the best candidates is also lower in DPM-HMM method than in LoopyMod. The DPM-HMM method produces all of its best candidates below 2.5 Å global RMSD, whereas LoopyMod has some cases upwards of 4 Å. In this canonical class, the DPM-HMM demonstrates that it performs well. Secondly, we tested the sampling efficiency of our method against LoopyMod for the non-canonical class of loops from CDRH3. The third and the fourth columns in Figure 8 show comparison of sampling efficiency for CDRH3 by both DPM-HMM and LoopyMod, respectively. As expected, the distribution from both methods is wider as compared to the RMSD distribution for canonical class of loops (CDRH1). The median global RMSD of the best candidates is lower from the DPM-HMM method. The best models have RMSDs of 0.77 Å and 1.05 Å by DPM-HMM and LoopyMod, respectively. The tighter distribution of the DPM-HMM for both the canonical and non-canonical class of loops indicates this method's ability to take advantage of the knowledge base in producing near native candidates. (See Figure S1 in Text S1 for scatter plot of individual data points)

## Discussion

### General Dependencies of the DPM-HMM Method

The DPM-HMM method's use of a knowledge base implies that the approach is dependent on quality of the input data. Because longer loops are sampled less accurately and less consistently, loop length needs to be included in the discussion. As Figures 4 and 8 show, the DPM-HMM method performs well with canonical loops, so the discussion will focus on the longer more difficult to predict loops. Loops with lengths of 15, 16 and 17 are modeled only with 2 to 8 templates. The longest 17 residue loops from CDRH3 was modeled with 8 templates and is considered to be a very difficult loop to model because of the length and the conformational variability (templates' average global RMSD is 7.43±0.24 Å). The best models for this group show average global RMSD of 3.13±0.32 Å, which improves upon the closest templates as well as models predicted by other methods for the loops of similar length [40], [44]. Recently, Choi *et. al.* [40] reported best models for 15–17 residue loops in the range of 3.48–4.75 Å. With best candidate global RMSDs between 1.12 to 1.81 Å (Figure 4), moderate length loops of 10–13 residues from CDRH3 were sampled closer to the target structure than that of the best templates. Another example of a longer loop used in this study is the 15 residue EF loop from the globin fold. There are only 3 EF loop targets with 15 residues; therefore one target is modeled using only the remaining two loops as input template data (average RMSD 3.11±1.45 Å). The best predicted candidates were found to deviate from the target structure on average by 2.43±0.39 Å. While many of the φ,ψ density estimations properly model the backbone torsion angles in high density areas (see Figures 2a and 2b), it requires only a few residues with angles reside in lower density regions of our density estimation (see Figures 2b and 2d) to make sampling a close model more difficult. It's also worth mentioning that two of the three globin EF loop 15mers are from crystal structures of the similar proteins with identical sequence in loop regions, however loop conformations varies from these 15mers with global RMSD of 2.26 Å. This reflects an extreme case of template based loop modeling with a limited number of templates, and the DPM-HMM method still achieves reasonable sampling efficiency in such difficult cases.

The templates' average global RMSD provides an independent measure about the variability within the input knowledge base that can be used in real-world conditions to predict model's sampling performance. Lower values of RMSDs result from similar loop conformations to the other templates and higher values are attributed to the large deviation of loop conformations in the template set. Hence, the wider the range of template average RMSDs, the more diverse the template set is. Yet, even with a large variability in conformational space, our DPM-HMM can sample a diverse φ,ψ distribution, and still produce models better than the best template. Even if templates' average RMSDs are larger than 7.0 Å, the best candidates are at a maximum 3.66 Å RMSD. One example of improvement is 17 residue CDRH3 loop from monoclonal antibody hGR-2 F6 (1dqd [44]). The 8 input templates possess an average template RMSD of 7.05 Å and the best template has the RMSD of 8.30 Å to the target structure (data point not shown in Figure 4). For this difficult case, the DPM-HMM method produced a best candidate with a 3.11 Å RMSD to the native loop. This result demonstrates the ability of the DMP-HMM method to produce consistently good models for even difficult loop modeling examples. A major reason for this ability is that the continuous density estimations do not outright exclude areas of Ramachandran space, but rather bias the more probable regions as informed by the input data from the templates. Unfortunately, the DPM-HMM method has a residue limit of about 20 amino acids as shown by our CASP9 results in Figure 7, where the method begins to under-sample the density estimations due to computational constraints. Overall, our approach directly addresses the familiar problem of insufficient templates as well as those all too common instances where the native loop uniquely deviates from the prevalent conformation of the templates at certain positions. For these reasons, the DPM-HMM method proves to be a reliable tool for loop modeling, since even with a small number of templates and low structural similarity, the DPM-HMM approach can quickly and thoroughly sample backbone φ,ψ space to identify loop structures near to native structure.

### Assessment of Method for Loop Modeling

In this study, we applied a novel loop modeling method, the Dirichlet process mixture of hidden Markov models or DPM-HMM [36], [37] for φ,ψ density estimation in loop regions. 465 target loops classified in 17 groups depending on the loop identity and length were modeled. These targets were representatives of the various challenges in loop modeling from the easier canonical loops to non-canonical loops with many residues and insufficient sampling. By estimating a continuous distribution across conformational space, the DPM-HMM method combines the advantages of continuous sampling from physical methods and propensities from knowledge-based methods without compromising modeling speed or being limited to specific conformations found in fragment-based libraries. The best global RMSD of a candidate is as low as 0.45 Å for one of the shortest loops (7 amino acid residues) from CDRH2. For these canonical loops with templates below 1.0 Å, the DPM-HMM produces improved models in about 38% of target loops. Also, It is also very encouraging that we can always improve the best templates when best template RMSDs are higher than 3.0 Å. For the most difficult case of a long 17 residue loop with sparse input data, the DPM-HMM approach produces models within 3.66 Å, which is the limit independent of loop length and quality of input data (Figures 4 and 5). Our results demonstrate that the DPM-HMM method provides consistent and reliable model sampling across the spectrum of loop modeling up to 20 residues.

The modeling accuracy was found to depend on three factors. The first, and most important, is loop length. It is well known that a loop becomes more difficult to model as length becomes longer (Figures 6a and 7), since more residues exponentially increase the potential conformational space. This quickly reduces the effective sampling that can be done. However, a loop with 17 amino acids was successfully modeled to 2.82 Å RMSD of the native with only 8 templates in the input data set. The low number of templates for input data points out the second factor: the number of templates available (Figure 5 and 6b). The sampling efficiency shows negative correlation with the number of templates when less than 30 templates are used (Figure 6b). The sampling efficiency becomes saturated when more than 30 templates are available. The last factor is the quality of the templates, which provides the input data for our density estimations. If near native templates are available, modeled loops are most likely close to the target structure. Even in cases where no good templates are available, the DPM-HMM method can produce improved loop models. Since all of the allowable Ramanchandran space possesses some probability in the density estimation, this approach can sample into underrepresented areas of conformational space and account for novel loop conformations outside of the representation of the knowledge base. To conclude, the DPM-HMM method can be generally applied as an effective and reliable template based loop-modeling algorithm as seen from the results for benchmarking loops from the CASP9 targets.

## Materials and Methods

### Data Sets

A dataset of 465 target loops was compiled for this study, as given in Table 1. For all loops, two anchoring residues on either side were included as anchoring residues. Structural alignments were performed using MUSTANG [45]. Structures of 132 immunoglobulin heavy variable domains at greater than 95% sequence identity were retrieved from the ASTRAL compendium of protein structure [46]. As one of the most common representatives for template-based loop modeling, the three complementarity-determining regions (CDRs) from the heavy chain were selected for modeling. According to IMGT numbering scheme [47], the CDR loop sets were constructed by extracting the residues at following sequence positions: 23–39 for CDRH1, 56–67 for CDRH2 and 104–118 for CDRH3. The second loop data set was taken from 92 globin structures which were downloaded from the PDB [48] and structurally aligned, as used previously by Tsai *et. al.* [36], [37]. DSSP [49] secondary structure profiles were used to determine the boundaries of the longest loop in the globin fold. This loop connecting helices E and F consists of alignment positions 93–106 in the multiple structural alignments. The third data set consisted of the templates for a CASP9 target, T0617 (3nrv). The longest loop containing 12 residues was extracted from 21 structurally superposed non-redundant template structures. Table S1 in Text S1 shows PDB identifiers and loop sequence positions for all the data sets used in this study.

Table 1 provides the details of the final data sets used and Figure 1 shows structural superposition of all the templates in each dataset. The set is briefly described here. A total of 352 well-defined loops from 132 antibody structures were classified into three major classes as CDRH1, CDRH2 and CDRH3. Not all the loop regions are well defined in each PDB, so each set consisted of slightly different numbers of templates. Therefore, 111 protein structures are in the CDRH1 loop set, which is well conserved with 12 residues in each loop structure. CDRH2 contains 130 loop structures and is subdivided in three groups by loop lengths of 7, 8 and 10 residue loops. CDRH3 is the most diverse dataset, containing a total of 111 loop structures that are grouped by sizes ranging from 8–17 amino acid residues. Next, 92 globin EF loops are grouped into the 3 classes: 12, 13, and 15 residue loops. Lastly, 21 target loops were extracted from the template structures to the CASP9 target T0617 and all loops are 12 residues in length. The crystal structure geometry of the backbone atoms (N, Cα and C) of the first anchoring residue (N-terminal) of the target loop was used to build the models. All the models were built starting from the second residue at N-terminal residue to the last residue at C-terminal. Sharing the first anchoring residue backbone coordinates results in globally superposed loops, so no further superposition is needed. Length of the loops refers to the total number of residues modeled. Although two more residues on both sides of the loop region are included in the sampling, only the second residue from the N-terminus and last two residues at the C-terminus were counted in the total number of residues (as defined by loop length).

### Dataset from CASP9 Targets

To show the general applicability of our sampling method outside a specific class or fold in protein family, data was compiled for 90 identified loops modeled during the CASP9 campaign. As this work focuses on loop sampling, only cases were considered where loop regions were identified correctly. For each target protein sequence from 305 putative loops in 45 TBM targets, closely similar templates were identified by a PSI-BLAST [50] search. Template structures were superposed by MUSTANG [45] program as described above. The target sequence was aligned to the multiple templates using the profile alignment function in Muscle [51]. Based on the multiple sequence/structure alignment of target sequence and templates, loop regions in the target sequence were defined. The loop region definition in some of the cases was erroneous depending on the quality and number of templates as well as accuracy of the sequence alignment. Also, loops with no available reference structures were excluded from this analysis. (See Table S2 in Text S1 for PDB ids of reference structures and positions of loops with their RMSDs).

### Comparison of Sampling Efficiency with Sampling Algorithm of LoopyMod

For fair comparisons with a common method for loop modeling, the dataset of canonical (CDRH1) and non-canonical (CDRH3) loop conformations were modeled using both DPM-HMM and LoopyMod program [13], [21]. Although LoopyMod is a complete loop prediction algorithm that includes sampling, scoring and ranking steps, we are interested in only comparing the sampling efficiency of our method to that of LoopyMod. Therefore, scoring and ranking steps in LoopyMod were omitted and all the sampled loop conformations were collected for global RMSD calculations. To simulate a realistic loop-modeling problem, the best template was provided as the input to the LoopyMod and a million conformations were generated. From these, a global backbone RMSDs against the reference crystal structure of the loop was calculated. (See Figure S1a and S1b in Text S1 for comparison of RMSD of best candidates to the RMSD of best template used by DPM-HMM and LoopyMod methods).

### Generation of Correlated φ,ψ Density Distributions

The joint φ,ψ distribution were estimated using the Dirichlet process mixture of hidden Markov models (DPM-HMM) [36], [37]. Data consists of sequences of angle pairs (φ*_ij_*, ψ*_ij_*), where *i* = 1,2,3,…,*n* is the index for a particular observed loop and *j* = 1,2,3,…,*m* is the index for the sequence position within the alignment. The model uses standard Bayesian nonparametrics density estimation techniques to estimate the joint density of all angle pairs across all *m* positions. Conceptually, it states that the data of loop *i* across the *m* positions - (φ*_i1_*,ψ*_i1_*), (φ*_i2_*, ψ*_i2_*),…, (φ*_im_*,ψ*_im_*) - arises from one of many clusters. Each cluster has a unique “centering” backbone angles, whereas members of a given cluster randomly deviate from its cluster center. The cluster to which each loop belongs is uncertain and the method mixes over this uncertainty, providing so-called mixture models. In contrast to many traditional mixture modeling approaches, however, the number of component distributions in our model is theoretically infinite, increasing the flexibility of the model.

Naively, one might simply model the “centering” backbone angles of each cluster as being independent, but that would ignore the obvious secondary structure that can readily be inferred from the observed data. Instead, the DPM-HMM considers a hidden Markov model for these “centering” backbone angles. Statistically, this represents a prior distribution on the values of parameters for the bivariate von Mises (BVM) sine model. The hidden states consisted of four secondary structure types: coil, helix, strand, and turn. The emission distributions for BVM location (or “centering”) parameters were bivariate von Mises sine model mixtures designed to mimic the distributions of torsion angles within each state from the PDB. Conditioning on inferred secondary structure (i.e., the hidden state in the Markov chain), the location parameters can be very specific. Transition probabilities among the states were also calculated based on observed distributions. Different emission distributions were used at locations containing proline or glycine due to the distinctive properties of these amino acids. (The emission distributions for scale parameters were identical for all states.) This informative centering distribution allowed us to leverage information along a sequence to provide informative secondary structure based density estimates even at positions with poor representation in an alignment.

Briefly, the formal statistical model can be written as:
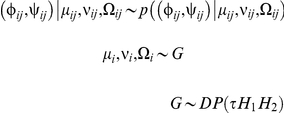
where *p* ((φ,ψ) | *μ*, ν, Ω) is a bivariate von Mises sine model [52] with mean parameters (*μ*,ν), *μ_i_* = *(μ_i1_, μ_i2_, μ_i3_, …, μ_im_)*, ν*_i_* = (ν*_i1_*, ν*_i2_*, ν*_i3_*, …, ν*_im_*), Ω*_i_* = (Ω*_i1_*, Ω*_i2_*, Ω*_i3_*, …, Ω*_im_*), and precision matrix Ω, *G* is a draw from a Dirichlet process with mass parameter *τ* and centering distributions *H_1_* for *μ_i_*, ν*_i_*, and *H_2_* for Ω*_i_*.. *H_2_* is taken to be the product of *m* identical Wishart distributions with shape parameter α_0_ and scale matrix β_0_, with an expected value of α_0_/(2β_0_). The distribution *H_1_* is the hidden Markov model discussed previously, with a state space consisting of four secondary structure classes (helix, turn, coil, and strand) each of which is represented by a mixture of between one and five bivariate von Mises sine models. A complete description of this method, including computational details, is provided in [36]. For each density estimate, we ran two Markov chain Monte Carlo (MCMC) [53] runs for 11,000 iterations with the first 1,000 discarded as burn in. Using 1-in-20 thinning, this gave us 1,000 draws from the posterior distribution, which forms the basis for our density estimate. For our hyperparameter settings, we took *τ = 5*. *H_2_* was the product of *m* independent Wishart distributions with shape parameter α_0_ = 2 and a 2×2 scale matrix β_0_, which had diagonal elements equal to 0.25 and off diagonal elements equal to 0. Because density estimation is the most computationally intensive portion of our loop-modeling scheme, this approach makes the simplification of only uniquely modeling positions with proline and glycine. For this reason, two loops can produce equivalent posterior distributions if their prolines and glycines appear in the same positions. Additional details on fitting this model and adjustments for gaps in alignment data are provided in previous work [36].

### DPM-HMM Model Building from φ,ψ Sample Space and Analysis

We used the leave one out (LOO) approach to model every target in a dataset. For each sampling and prediction run, the target loop is left out: not included as input data for the DPM-HMM density estimation. Remaining loops from the subgroup of the target were then used as templates to model and sample the joint φ,ψ distributions for a target sequence. A set of one million φ,ψ draws from the estimated densities was generated for each of the target loops. For all the one million draws of the torsion angles, all backbone atom models were constructed in Cartesian coordinate space using Self-Normalizing Natural Extension Reference Frame (SNerf) algorithm [54] with standard bond length and angle data [52]. In comparison to the initial density estimation, these two steps of making draws from the distribution and building the loop in Cartesian coordinates are relatively fast. About three hours of CPU time are required to sample one million points in φ,ψ space for an average sized target loop (about 12 amino acid residues). Model building from the sampled torsion angle space and filtering using average backbone α carbon (Cα) distance takes about 1.5 hours of CPU time. The computational expense scales linearly with the number of residues in the loop and the number of models to be built.

Three backbone atom coordinates of the first residue at entering N-terminal side of the target loop were used as the anchor for construction of the models in a Cartesian space. So all models in the set are built from the same starting point. To ensure appropriate loop closure, models were refined using a simple distance filter with a 2.0 Å cutoff value. This Cα distance filter is very basic using the average distance between the last two Cα atoms of a candidate loop model and those of target loop crystal structure in the loop exit. Since the backbone atom coordinates of first anchoring residue are shared in all the models and the reference structure, this simple filter works well and produces a pool of suitable candidates. Filtered models can be further scored for side chain clashes after grafting on the surface of the whole protein.

The DPM-HMM software used in this analysis is available for download at http://www.stat.tamu.edu/~dahl/software/cortorgles/.

## Supporting Information

Text S1 Primary data for the five classes of loops used for statistical modeling and sampling (Table S1a through S1e) and data for target loops that were modeled during CASP9 experiment (Table S2). Figure S1 shows comparison of RMSDs for the best candidates modeled using DPM-HMM and LoopyMod methods for (a) canonical and (b) non-canonical classes of loops.
(DOC)
