# Supplementary material for: Near-Native Protein Loop Sampling Using Nonparametric Density Estimation Accommodating Sparcity
Source: PLoS Comput Biol. 2011 Oct 20;7(10):e1002234. doi: 10.1371/journal.pcbi.1002234 (PMC3197639; doi:10.1371/journal.pcbi.1002234)
Supplement: Text S1 — Primary data for the five classes of loops used for statistical modeling and sampling (Table S1a through S1e) and data for target loops that were modeled during CASP9 experiment (Table S2). Figure S1 shows comparison of RMSDs for the best candidates modeled using DPM-HMM and LoopyMod methods for (a) canonical and (b) non-canonical classes of loops. (DOC) [file pcbi.1002234.s001.doc]

**Text S1**

Primary data for the five classes of loops used for statistical modeling and sampling (Table S1a through S1e) and data for target loops that were modeled during CASP9 experiment (Table S2). Figure S1 shows comparison of RMSDs for the best candidates modeled using DPM-HMM and LoopyMod methods for (a) canonical and (b) non-canonical classes of loops.

**Table S1a**

| **PDB ID** | **Loop Positions** | | **Best Candidate RMSD (Å)** |
| --- | --- | --- | --- |
|  | **Start** | **End** |  |
| 1a0qh | 22 | 34 | 0.91 |
| 1a3rh | 22 | 34 | 0.98 |
| 1a5fh | 22 | 34 | 1.22 |
| 1adqh | 22 | 34 | 0.88 |
| 1ae6h | 22 | 34 | 1.07 |
| 1afvh | 22 | 34 | 1.12 |
| 1aj7h | 22 | 34 | 1.49 |
| 1aqkh | 22 | 34 | 0.91 |
| 1axth | 22 | 34 | 0.89 |
| 1b2wh | 22 | 34 | 2.53 |
| 1b4jh | 22 | 34 | 1.56 |
| 1bj1h | 22 | 34 | 1.24 |
| 1bm3h | 22 | 34 | 1.07 |
| 1bqlh | 22 | 34 | 1.15 |
| 1c1eh | 22 | 34 | 0.88 |
| 1c5ch | 22 | 34 | 0.97 |
| 1ce1h | 22 | 34 | 1.07 |
| 1cloh | 22 | 34 | 0.86 |
| 1cr9h | 22 | 34 | 1.18 |
| 1d5ih | 22 | 34 | 1.05 |
| 1dbbh | 22 | 34 | 0.99 |
| 1dfbh | 22 | 34 | 1.27 |
| 1dqdh | 22 | 34 | 1.18 |
| 1e4wh | 22 | 34 | 1.19 |
| 1e6oh | 22 | 34 | 0.94 |
| 1egjh | 22 | 34 | 0.95 |
| 1ejoh | 21 | 33 | 0.97 |
| 1emth | 22 | 34 | 0.93 |
| 1eo8h | 22 | 34 | 0.82 |
| 1f4xh | 22 | 34 | 0.84 |
| 1faih | 22 | 34 | 1.24 |
| 1fbih | 22 | 34 | 1.04 |
| 1fe8h | 22 | 34 | 0.82 |
| 1fgnh | 22 | 34 | 0.98 |
| 1fh5h | 19 | 31 | 0.78 |
| 1figh | 22 | 34 | 1.55 |
| 1flrh | 22 | 34 | 0.91 |
| 1fnsh | 236 | 248 | 0.90 |
| 1forh | 22 | 34 | 1.14 |
| 1fpth | 22 | 34 | 0.99 |
| 1frgh | 239 | 251 | 1.07 |
| 1gafh | 22 | 34 | 1.75 |
| 1gpoh | 22 | 34 | 1.07 |
| 1h3ph | 22 | 34 | 1.26 |
| 1i9rh | 22 | 34 | 0.96 |
| 1ibgh | 22 | 34 | 1.08 |
| 1igch | 22 | 34 | 1.07 |
| 1igfh | 22 | 34 | 1.43 |
| 1ikfh | 22 | 34 | 0.94 |
| 1indh | 22 | 34 | 0.89 |
| 1iqwh | 22 | 34 | 0.90 |
| 1it9h | 22 | 34 | 0.84 |
| 1jfqh | 322 | 334 | 0.95 |
| 1jguh | 22 | 34 | 1.27 |
| 1jnlh | 22 | 34 | 0.95 |
| 1jpth | 22 | 34 | 0.82 |
| 1k6qh | 22 | 34 | 0.86 |
| 1kb5h | 22 | 34 | 1.20 |
| 1kegh | 22 | 34 | 0.87 |
| 1kemh | 22 | 34 | 0.66 |
| 1kfah | 22 | 34 | 0.87 |
| 1l7ih | 22 | 34 | 1.02 |
| 1l7th | 22 | 34 | 0.96 |
| 1lk3h | 22 | 34 | 0.77 |
| 1lo0h | 22 | 34 | 1.02 |
| 1lo4h | 22 | 34 | 1.18 |
| 1mamh | 22 | 34 | 0.88 |
| 1mcph | 22 | 34 | 0.89 |
| 1mexh | 22 | 34 | 0.94 |
| 1mhph | 22 | 34 | 1.01 |
| 1mimh | 20 | 32 | 1.00 |
| 1mj8h | 22 | 34 | 1.53 |
| 1mjuh | 22 | 34 | 0.90 |
| 1mreh | 22 | 34 | 1.07 |
| 1n0xh | 22 | 34 | 0.78 |
| 1nakh | 22 | 34 | 0.94 |
| 1nbvh | 22 | 34 | 1.51 |
| 1ncbh | 22 | 34 | 1.00 |
| 1nl0h | 22 | 34 | 0.89 |
| 1nlbh | 22 | 34 | 0.84 |
| 1nldh | 22 | 34 | 1.16 |
| 1op3h | 22 | 34 | 1.29 |
| 1osph | 22 | 34 | 1.32 |
| 1plgh | 22 | 34 | 0.76 |
| 1pskh | 22 | 34 | 0.83 |
| 1q0xh | 22 | 34 | 0.79 |
| 1q1jh | 22 | 34 | 1.05 |
| 1q72h | 22 | 34 | 0.79 |
| 1qfuh | 22 | 34 | 0.90 |
| 1qkzh | 22 | 34 | 0.84 |
| 1rihh | 22 | 34 | 0.96 |
| 1rmfh | 22 | 34 | 0.95 |
| 1rurh | 22 | 34 | 0.93 |
| 1rz7h | 22 | 34 | 0.85 |
| 1rzfh | 22 | 34 | 1.59 |
| 1s3kh | 22 | 34 | 0.92 |
| 1sbsh | 22 | 34 | 0.92 |
| 1sm3h | 22 | 34 | 0.91 |
| 1sy6h | 22 | 34 | 0.61 |
| 1teth | 22 | 34 | 1.19 |
| 1u6ah | 22 | 34 | 1.25 |
| 1ucbh | 22 | 34 | 0.91 |
| 1um5h | 23 | 35 | 2.27 |
| 1uweh | 22 | 34 | 0.97 |
| 1uwgh | 22 | 34 | 1.38 |
| 1v7mh | 22 | 34 | 0.98 |
| 1vgeh | 23 | 35 | 0.98 |
| 1w72h | 22 | 34 | 0.84 |
| 1wejh | 22 | 34 | 0.79 |
| 1yeeh | 22 | 34 | 0.80 |
| 1yeih | 22 | 34 | 0.86 |
| **Average** |  | | **1.04** |
| **Median** |  |  | **0.96** |

**Table S1b**

| **PDB ID** | **Loop Positions** | | **Best Candidate RMSD (Å)** |
| --- | --- | --- | --- |
|  | **Start** | **End** |  |
| **10 Residue Loop** | | | |
| 1axth | 51 | 58 | 0.73 |
| 1ce1h | 51 | 61 | 0.94 |
| 1cloh | 51 | 58 | 0.81 |
| 1flrh | 51 | 61 | 0.80 |
| 1kemh | 51 | 61 | 0.86 |
| 1mamh | 51 | 61 | 1.11 |
| 1mcph | 51 | 61 | 1.05 |
| 1nbvh | 51 | 61 | 0.72 |
| 1q1jh | 51 | 58 | 0.74 |
| 1q72h | 51 | 58 | 0.76 |
| 1sbsh | 51 | 61 | 0.50 |
| 1sm3h | 51 | 58 | 0.75 |
| 1v7mh | 51 | 61 | 0.80 |
| **Average** |  | | **0.81** |
| **Median** |  |  | **0.80** |
|  | | | |
| **7 Residue Loop** | | | |
| 1ai1h | 51 | 58 | 0.52 |
| 1bafh | 52 | 59 | 0.65 |
| 1cf8h | 51 | 58 | 0.54 |
| 1dqdh | 51 | 58 | 0.60 |
| 1f58h | 51 | 58 | 0.57 |
| 1f8th | 51 | 58 | 0.58 |
| 1fe8h | 51 | 58 | 0.67 |
| 1fnsh | 265 | 272 | 0.58 |
| 1ggbh | 51 | 58 | 0.57 |
| 1gigh | 51 | 58 | 0.45 |
| 1gpoh | 51 | 58 | 0.49 |
| 1ibgh | 51 | 58 | 0.58 |
| 1jguh | 51 | 58 | 1.42 |
| 1jrhh | 51 | 58 | 0.52 |
| 1kcrh | 52 | 59 | 0.49 |
| 1kcuh | 52 | 59 | 0.49 |
| 1kcvh | 52 | 59 | 0.54 |
| 1kfah | 51 | 59 | 0.58 |
| 1l7th | 51 | 58 | 0.48 |
| 1lo0h | 51 | 61 | 0.57 |
| 1lo4h | 51 | 58 | 0.52 |
| 1mhph | 51 | 58 | 0.64 |
| 1nakh | 51 | 58 | 0.61 |
| 1ncwh | 51 | 58 | 0.59 |
| 1nldh | 51 | 58 | 0.52 |
| 1nsnh | 51 | 58 | 0.68 |
| 1osph | 51 | 58 | 1.13 |
| 1r0ah | 53 | 60 | 0.76 |
| 1tjgh | 51 | 58 | 0.68 |
| 1u6ah | 51 | 58 | 0.68 |
| **Average** |  | | **0.62** |
| **Median** |  |  | **0.58** |
|  | | | |
| **8 Residue Loop** | | | |
| 1a0qh | 51 | 58 | 0.91 |
| 1a3rh | 51 | 58 | 0.58 |
| 1a5fh | 51 | 58 | 0.97 |
| 1adqh | 51 | 58 | 0.67 |
| 1ae6h | 51 | 58 | 0.90 |
| 1afvh | 51 | 58 | 0.70 |
| 1aj7h | 51 | 58 | 0.84 |
| 1aqkh | 51 | 58 | 0.80 |
| 1ay1h | 51 | 58 | 1.35 |
| 1b2wh | 51 | 58 | 0.74 |
| 1b4jh | 51 | 58 | 0.81 |
| 1bj1h | 51 | 58 | 0.67 |
| 1bm3h | 51 | 58 | 0.74 |
| 1bqlh | 51 | 58 | 1.07 |
| 1c1eh | 51 | 58 | 0.79 |
| 1c5ch | 51 | 58 | 0.63 |
| 1cr9h | 51 | 58 | 0.92 |
| 1d5ih | 51 | 58 | 1.16 |
| 1dbbh | 51 | 58 | 0.69 |
| 1dfbh | 51 | 58 | 0.73 |
| 1e4wh | 51 | 58 | 0.70 |
| 1e6oh | 51 | 58 | 0.80 |
| 1egjh | 51 | 58 | 0.70 |
| 1ejoh | 50 | 58 | 0.63 |
| 1emth | 51 | 58 | 1.03 |
| 1eo8h | 51 | 58 | 1.09 |
| 1f3dh | 51 | 58 | 0.84 |
| 1f4xh | 51 | 58 | 0.82 |
| 1faih | 51 | 58 | 0.92 |
| 1fbih | 51 | 58 | 0.84 |
| 1fgnh | 51 | 58 | 0.84 |
| 1fh5h | 48 | 55 | 0.71 |
| 1figh | 51 | 58 | 1.13 |
| 1forh | 51 | 58 | 0.85 |
| 1fpth | 51 | 58 | 0.94 |
| 1frgh | 268 | 275 | 0.75 |
| 1gafh | 51 | 58 | 0.90 |
| 1ghfh | 50 | 57 | 0.65 |
| 1h3ph | 51 | 58 | 0.79 |
| 1i9rh | 51 | 58 | 0.84 |
| 1igch | 51 | 58 | 0.70 |
| 1igfh | 51 | 58 | 0.72 |
| 1ikfh | 51 | 58 | 0.68 |
| 1indh | 51 | 58 | 1.32 |
| 1iqwh | 51 | 58 | 0.81 |
| 1it9h | 51 | 58 | 0.90 |
| 1jfqh | 351 | 358 | 0.77 |
| 1jglh | 51 | 58 | 0.62 |
| 1jnlh | 51 | 58 | 1.06 |
| 1jpth | 51 | 58 | 0.96 |
| 1k6qh | 51 | 58 | 0.84 |
| 1kb5h | 51 | 58 | 0.74 |
| 1kegh | 51 | 58 | 0.69 |
| 1kenh | 52 | 59 | 1.49 |
| 1l7ih | 51 | 58 | 0.75 |
| 1lk3h | 51 | 58 | 0.69 |
| 1mexh | 51 | 58 | 0.92 |
| 1mimh | 49 | 56 | 1.04 |
| 1mj8h | 51 | 58 | 0.88 |
| 1mjuh | 51 | 58 | 1.01 |
| 1mreh | 51 | 58 | 0.54 |
| 1n0xh | 51 | 58 | 0.77 |
| 1ncbh | 51 | 58 | 0.73 |
| 1nl0h | 51 | 58 | 0.67 |
| 1nlbh | 51 | 58 | 0.72 |
| 1op3h | 51 | 58 | 1.15 |
| 1plgh | 51 | 58 | 1.04 |
| 1pskh | 51 | 58 | 1.06 |
| 1q0xh | 51 | 58 | 0.82 |
| 1qfuh | 51 | 58 | 0.79 |
| 1qkzh | 51 | 58 | 0.71 |
| 1rihh | 51 | 58 | 0.73 |
| 1rurh | 51 | 58 | 0.97 |
| 1rz7h | 51 | 58 | 0.93 |
| 1rzfh | 51 | 58 | 0.82 |
| 1s3kh | 51 | 58 | 1.17 |
| 1sy6h | 51 | 58 | 0.68 |
| 1teth | 51 | 58 | 0.62 |
| 1ucbh | 51 | 58 | 0.60 |
| 1um5h | 51 | 58 | 0.73 |
| 1uweh | 51 | 58 | 1.31 |
| 1uwgh | 51 | 58 | 1.14 |
| 1vgeh | 52 | 59 | 0.66 |
| 1w72h | 51 | 58 | 0.67 |
| 1wejh | 51 | 58 | 0.90 |
| 1yeeh | 51 | 58 | 0.86 |
| 1yeih | 51 | 58 | 0.67 |
| **Average** |  | | **0.84** |
| **Median** |  |  | **0.81** |

Table S1c

| **PDB ID** | **Loop Positions** | | **Best Candidate RMSD (Å)** |
| --- | --- | --- | --- |
|  | **Start** | **End** |  |
| **10 Residue Loops** | | | |
| 1axth | 92 | 103 | 1.17 |
| 1bqlh | 96 | 106 | 1.46 |
| 1cf8h | 92 | 103 | 1.61 |
| 1fe8h | 95 | 105 | 1.43 |
| 1flrh | 98 | 108 | 1.53 |
| 1ghfh | 95 | 105 | 1.72 |
| 1h3ph | 92 | 103 | 1.70 |
| 1jglh | 92 | 103 | 1.33 |
| 1kcrh | 92 | 106 | 1.12 |
| 1kcuh | 96 | 106 | 1.56 |
| 1kcvh | 96 | 106 | 1.72 |
| 1mjuh | 92 | 103 | 1.27 |
| 1mreh | 92 | 103 | 1.36 |
| 1n7mh | 88 | 98 | 1.77 |
| 1teth | 92 | 103 | 1.20 |
| **Average** |  | | **1.46** |
| **Median** |  |  | **1.46** |
|  | | | |
| **11 Residue Loops** | | | |
| 1b2wh | 96 | 107 | 2.12 |
| 1b4jh | 96 | 107 | 1.20 |
| 1f4xh | 96 | 107 | 1.52 |
| 1fgnh | 96 | 107 | 1.33 |
| 1jpth | 96 | 107 | 1.18 |
| 1k6qh | 96 | 107 | 1.46 |
| 1kemh | 98 | 109 | 1.71 |
| 1mamh | 98 | 109 | 1.96 |
| 1mimh | 94 | 105 | 1.17 |
| 1plgh | 96 | 107 | 1.55 |
| 1uweh | 95 | 103 | 1.60 |
| 1uwgh | 95 | 103 | 1.32 |
| 1wejh | 96 | 107 | 1.68 |
| **Average** |  | | **1.52** |
| **Median** |  |  | **1.52** |
| **12 Residue Loops** | | | |
| 1a3rh | 92 | 103 | 2.17 |
| 1eo8h | 92 | 103 | 1.99 |
| 1f8th | 92 | 103 | 1.81 |
| 1i9rh | 96 | 108 | 2.18 |
| 1nlbh | 92 | 103 | 2.21 |
| 1um5h | 97 | 109 | 1.64 |
| **Average** |  | | **2.10** |
| **Median** |  |  | **2.17** |
|  | | | |
| **13 Residue Loops** | | | |
| 1ay1h | 92 | 105 | 1.56 |
| 1c1eh | 92 | 103 | 1.32 |
| 1ce1h | 98 | 111 | 2.20 |
| 1cloh | 92 | 103 | 3.53 |
| 1dbbh | 92 | 103 | 1.78 |
| 1egjh | 92 | 103 | 1.53 |
| 1figh | 92 | 103 | 1.72 |
| 1forh | 96 | 109 | 1.80 |
| 1igfh | 92 | 103 | 2.20 |
| 1jguh | 92 | 103 | 1.79 |
| 1kb5h | 92 | 103 | 1.54 |
| 1l7ih | 92 | 103 | 1.80 |
| 1l7th | 95 | 108 | 1.84 |
| 1lk3h | 96 | 109 | 1.97 |
| 1lo0h | 98 | 109 | 2.16 |
| 1lo4h | 95 | 108 | 1.87 |
| 1mhph | 95 | 108 | 2.13 |
| 1mj8h | 92 | 103 | 2.11 |
| 1nbvh | 98 | 111 | 2.22 |
| 1ncwh | 92 | 103 | 1.78 |
| 1q0xh | 92 | 103 | 1.59 |
| 1qkzh | 92 | 103 | 1.80 |
| 1rmfh | 96 | 109 | 1.87 |
| 1rurh | 92 | 103 | 2.21 |
| 1rz7h | 92 | 103 | 2.03 |
| 1s3kh | 96 | 109 | 2.17 |
| 1sy6h | 96 | 109 | 1.71 |
| 1ucbh | 92 | 103 | 1.66 |
| **Average** |  | | **1.92** |
| **Median** |  |  | **1.82** |
|  | | | |
| **14 Residue Loops** | | | |
| 1ae6h | 92 | 103 | 1.81 |
| 1afvh | 96 | 110 | 2.07 |
| 1ai1h | 92 | 103 | 1.76 |
| 1d5ih | 92 | 103 | 2.56 |
| 1e6oh | 96 | 110 | 2.22 |
| 1ejoh | 95 | 109 | 2.06 |
| 1fpth | 92 | 103 | 2.11 |
| 1frgh | 313 | 327 | 2.19 |
| 1igch | 96 | 110 | 1.92 |
| 1kegh | 92 | 103 | 2.01 |
| 1kenh | 96 | 110 | 3.24 |
| 1mcph | 98 | 112 | 1.72 |
| 1ncbh | 92 | 103 | 2.12 |
| 1nl0h | 92 | 103 | 2.43 |
| **Average** |  | | **2.16** |
| **Median** |  |  | **2.09** |
|  | | | |
| **15 Residue Loops** | | | |
| 1iqwh | 96 | 111 | 1.64 |
| 1it9h | 96 | 111 | 2.19 |
| 1jfqh | 396 | 411 | 2.56 |
| 1jrhh | 92 | 103 | 2.11 |
| 1kfah | 96 | 111 | 2.30 |
| 1osph | 95 | 110 | 2.70 |
| 1sbsh | 98 | 113 | 2.22 |
| 1vgeh | 97 | 112 | 3.19 |
| **Average** |  | | **2.36** |
| **Median** |  |  | **2.26** |
|  | | | |
| **16 Residue Loops** | | | |
| 1fbih | 96 | 112 | 2.92 |
| 1ibgh | 92 | 103 | 3.04 |
| 1qfuh | 92 | 103 | 3.37 |
| 1r0ah | 97 | 113 | 3.08 |
| 1w72h | 92 | 103 | 3.33 |
| **Average** |  | | **3.15** |
| **Median** |  |  | **3.08** |
|  | | | |
| **17 Residue Loops** | | | |
| 1adqh | 92 | 103 | 2.82 |
| 1aqkh | 96 | 113 | 3.49 |
| 1bj1h | 96 | 113 | 2.84 |
| 1dqdh | 95 | 112 | 3.11 |
| 1fnsh | 309 | 326 | 3.08 |
| 1gigh | 95 | 112 | 3.42 |
| 1op3h | 92 | 103 | 3.66 |
| 1yeeh | 92 | 103 | 2.84 |
| 1yeih | 92 | 103 | 2.95 |
| **Average** |  | | **3.13** |
| **Median** |  |  | **3.08** |
|  | | | |
| **8 Residue Loops** | | | |
| 1aj7h | 95 | 103 | 0.87 |
| 1cr9h | 92 | 100 | 1.28 |
| 1e4wh | 92 | 100 | 1.10 |
| 1gafh | 96 | 104 | 0.77 |
| 1ggbh | 92 | 103 | 1.22 |
| 1gpoh | 95 | 103 | 1.18 |
| 1indh | 96 | 104 | 1.04 |
| 1jnlh | 96 | 104 | 1.29 |
| 1mexh | 92 | 103 | 1.30 |
| 1nakh | 92 | 103 | 1.01 |
| 1nldh | 92 | 103 | 1.15 |
| 1pskh | 96 | 104 | 0.96 |
| 1v7mh | 98 | 106 | 0.89 |
| **Average** |  | | **1.08** |
| **Median** |  |  | **1.10** |

**Table S1d**

| **PDB ID** | **Loop Positions** | | **Best Candidate RMSD (Å)** |
| --- | --- | --- | --- |
|  | **Start** | **End** |  |
| **12 Residue Loops** | | | |
| 1b0b | 95 | 107 | 0.82 |
| 1binA | 92 | 104 | 1.52 |
| 1buwB | 92 | 104 | 1.66 |
| 1cg5B | 88 | 100 | 0.79 |
| 1cqxA | 85 | 97 | 0.84 |
| 1d8uA | 108 | 120 | 1.24 |
| 1dm1 | 95 | 107 | 0.95 |
| 1ebt | 96 | 108 | 0.81 |
| 1eca | 87 | 99 | 0.94 |
| 1flp | 96 | 108 | 0.77 |
| 1gdi | 96 | 109 | 1.59 |
| 1gvh | 85 | 97 | 0.58 |
| 1h97A | 98 | 110 | 1.05 |
| 1hbiA | 100 | 112 | 1.07 |
| 1hlb | 104 | 116 | 0.72 |
| 1hlm | 104 | 116 | 1.03 |
| 1ithA | 94 | 106 | 0.71 |
| 1mba | 95 | 107 | 0.72 |
| 1sctA | 101 | 113 | 0.95 |
| 1sctB | 102 | 114 | 0.91 |
| 2vhbA | 76 | 88 | 1.01 |
| 2vhbB | 77 | 89 | 1.07 |
| 4vhbA | 77 | 89 | 0.88 |
| **Average** |  | | **0.98** |
| **Median** |  |  | **0.94** |
|  | | | |
| **13 Residue Loops** | | | |
| 101m | 94 | 107 | 0.79 |
| 104m | 93 | 106 | 0.94 |
| 1a00A | 87 | 100 | 1.34 |
| 1a00B | 92 | 105 | 1.29 |
| 1a3nB | 91 | 104 | 1.04 |
| 1a4fA | 87 | 100 | 1.07 |
| 1a4fB | 92 | 105 | 0.82 |
| 1a6g | 93 | 106 | 1.11 |
| 1a9wE | 92 | 105 | 1.14 |
| 1abwA | 87 | 100 | 0.87 |
| 1axfB | 92 | 105 | 1.18 |
| 1azi | 93 | 106 | 0.96 |
| 1babA | 88 | 101 | 1.01 |
| 1c7dA | 88 | 101 | 0.91 |
| 1cg5A | 88 | 101 | 0.84 |
| 1ch4A | 92 | 105 | 1.02 |
| 1dshA | 87 | 100 | 0.68 |
| 1dtm | 93 | 106 | 1.17 |
| 1dwr | 93 | 106 | 1.22 |
| 1dxtB | 93 | 106 | 1.07 |
| 1dxvB | 92 | 105 | 1.11 |
| 1emy | 93 | 106 | 1.04 |
| 1f5oA | 105 | 118 | 1.25 |
| 1fawA | 87 | 100 | 1.14 |
| 1fdhG | 92 | 105 | 1.16 |
| 1fhjA | 87 | 100 | 0.97 |
| 1fhjB | 92 | 105 | 1.06 |
| 1fsxA | 87 | 100 | 1.01 |
| 1fsxB | 91 | 104 | 0.89 |
| 1g0bA | 87 | 100 | 1.05 |
| 1g0bB | 92 | 105 | 1.10 |
| 1gcvA | 87 | 100 | 1.13 |
| 1gcvB | 82 | 95 | 1.11 |
| 1gcwB | 78 | 91 | 1.15 |
| 1hbhA | 88 | 101 | 1.30 |
| 1hbhB | 92 | 105 | 1.16 |
| 1hbrA | 87 | 100 | 1.11 |
| 1hbrB | 92 | 105 | 1.05 |
| 1hbrC | 87 | 100 | 1.16 |
| 1hbrD | 92 | 105 | 1.39 |
| 1hdsA | 87 | 100 | 1.06 |
| 1hdsB | 91 | 104 | 0.98 |
| 1it2A | 103 | 116 | 0.99 |
| 1jebA | 87 | 100 | 1.03 |
| 1jebB | 92 | 105 | 1.25 |
| 1jebD | 92 | 105 | 0.94 |
| 1la6A | 88 | 101 | 0.97 |
| 1la6B | 81 | 94 | 0.95 |
| 1lhs | 93 | 106 | 1.39 |
| 1m6cA | 93 | 106 | 1.03 |
| 1mbs | 93 | 106 | 1.12 |
| 1mnjB | 93 | 106 | 1.23 |
| 1myt | 88 | 101 | 1.10 |
| 1o1kA | 86 | 99 | 1.09 |
| 1outA | 88 | 101 | 1.25 |
| 1outB | 92 | 105 | 1.14 |
| 1qpwA | 87 | 100 | 1.05 |
| 1qpwB | 92 | 105 | 1.11 |
| 1spgA | 89 | 102 | 0.68 |
| 1spgB | 92 | 105 | 1.32 |
| 1t1nB | 92 | 105 | 1.14 |
| 1uc3A | 105 | 118 | 1.03 |
| 2dhbB | 92 | 105 | 1.13 |
| 2hheB | 91 | 104 | 1.24 |
| 2mm1 | 93 | 106 | 1.13 |
| 2lhb | 105 | 118 | 1.17 |
| **Average** |  | | **1.08** |
| **Median** |  |  | **1.10** |
|  | | | |
| **15 Residue Loops** | | | |
| 1hbg | 90 | 105 | 2.77 |
| 1jf3 | 90 | 105 | 2.01 |
| 1jf4 | 90 | 105 | 2.51 |
| **Average** |  |  | **2.43** |
| **Median** |  |  | **2.51** |

Table S1e

| **PDB ID** | **Loop Positions** | | **Best Candidate RMSD (Å)** |
| --- | --- | --- | --- |
|  | **Start** | **End** |  |
| **12 Residue Loops** | | | |
| 1jgsa | 86 | 98 | 1.52 |
| 1lj9a | 76 | 88 | 1.10 |
| 1lnwa | 83 | 95 | 1.37 |
| 1s3ja | 83 | 95 | 0.96 |
| 1z91a | 86 | 98 | 1.00 |
| 2a61a | 80 | 92 | 0.94 |
| 2bv6a | 84 | 96 | 1.03 |
| 2etha | 78 | 90 | 0.97 |
| 2fbha | 82 | 94 | 2.26 |
| 2fbia | 80 | 92 | 0.88 |
| 2nnna | 84 | 96 | 1.11 |
| 2nyxa | 91 | 103 | 1.44 |
| 3bdda | 76 | 88 | 0.99 |
| 3bj6a | 83 | 95 | 1.07 |
| 3bjaa | 78 | 90 | 1.09 |
| 3bpxa | 79 | 91 | 0.77 |
| 3f3xa | 82 | 94 | 1.30 |
| 3gfja | 82 | 94 | 1.27 |
| 3gfla | 82 | 94 | 1.27 |
| 3gfma | 82 | 94 | 1.32 |
| 3k0la | 89 | 101 | 2.24 |
| **Average** |  | | **1.23** |
| **Median** |  |  | **1.10** |

**Table S2**

| **CASP9 Target** | | **Reference**  **PDB** | **Loop Sequence**  **(Loop Boundaries)** | **Candidate Global RMSD (Å)** | | | **Length (AA)** |
| --- | --- | --- | --- | --- | --- | --- | --- |
| 1 | T0520 | 3mr7 | VLEDGDI  (103-109) | 0.592 | | | 7 |
|  | | | ISEPGAI  (123-129) | 0.34 | | | 7 |
|  | | | | | | | |
| 2 | T0543 | 2xrg | VDGFRAS  (170-176) | 0.608 | | | 7 |
|  | | | KKGSKVM  (179-185) | 0.882 | | | 7 |
|  |  |  | THAPYMRPVYPTKT  (196-209) | 2.75 | | | 14 |
|  |  |  | SHGIVGNS  (224-231) | 0.773 | | | 8 |
|  |  |  | FNHRWWGGQP  (249-258) | 1.448 | | | 10 |
|  |  |  | LPDNERPSVY  (294-303) | 1.871 | | | 10 |
|  |  |  | FVGYGPTFKYRTK  (487-499) | 2.297 | | | 13 |
|  |  |  | KVPPFEN  (499-505) | 0.48 | | | 7 |
|  | | | | | | | |
| 3 | T0547 | 3nzp | LLEQF  (223-227) | 0.489 | | | 5 |
|  | | | LFDLGYVDL  (391-399) | 2.062 | | | 9 |
|  |  |  | IISINEK  (537-543) | 0.863 | | | 7 |
|  | | | | | | | |
| 4 | T0563 | 3on7 | YYHVYPWGRIP  (88-98) | 2.294 | | | 11 |
|  | | | FSIPLPEM  (135-142) | 1.86 | | | 8 |
|  |  |  | IANSHKT  (143-149) | 1.251 | | | 7 |
|  | | | | | | | |
| 5 | T0565 | 3npf | HISVC  (37-41) | 0.624 | | | 4 |
|  | | | QYNGWYE  (67-73) | 1.517 | | | 6 |
|  |  |  | VTSHYGFA  (108-115) | 1.052 | | | 7 |
|  |  |  | DVVAGNR  (128-134) | 0.68 | | | 6 |
|  |  |  | MMGIPYLWAGTSSKGVDC  (186-203) | 3.821 | | | 17 |
|  |  |  | FFGRKATAERKEGIS  (248-262) | 2.634 | | | 14 |
|  | | | | | | | |
| 6 | T0570 | 3no3 | EIGAYGSE  (52-59) | 0.514 | | | 8 |
|  | | | YHDNDIQGKHIQSCT  (73-87) | 2.198 | | | 15 |
|  |  |  | LNGELS  (178-183) | 1.114 | | | 6 |
|  | | | | | | | |
| 7 | T0573 | 3oox | DLPPGHRFRAHMADNVWPAEIPA  (103-125) | 5.895 | | | 22 |
|  | | | LLGAEEGGLE  (199-208) | 1.665 | | | 9 |
|  | | | | | | | |
| 8 | T0575 | 3nrg | RAGIAKGSF  (41-49) | 0.91 | | | 8 |
|  | | | | | | | |
| 9 | T0585 | 3ne8 | QKGSHTI  (219-225) | 1.209 | | | 7 |
|  | | | YTISDEASD  (272-280) | 1.656 | | | 9 |
|  | | | | | | | |
| 10 | T0586 | 3neu | KRGMGSF  (67-73) | 5.419 | | | 7 |
|  | | | | | | | |
| 11 | T0589 | 3net | NIGDFV  (173-178) | 1.121 | | | 6 |
|  | | | AIARGLNYY  (295-303) | 1.614 | | | 9 |
|  | | | | | | | |
| 12 | T0591 | 3nra | RYGAT  (209-213) | 0.246 | | | 5 |
|  | | | | | | | |
| 13 | T0592 | 3nhv | KKGYEGII  (43-50) | 1.314 | | | 8 |
|  | | | YCWGPAC  (93-99) | 0.678 | | | 7 |
|  | | | | | | | |
| 14 | T0593 | 3ngw | KYSPFQT  (35-41) | 1.084 | | | 7 |
|  | | | KAGCDAL  (110-116) | 1.217 | | | 7 |
|  |  |  | AILQGIR  (141-147) | 1.041 | | | 7 |
|  | | | | | | | |
| 15 | T0594 | 3ni8 | SLFSGSI  (64-70) | 0.673 | | | 7 |
|  | | | KFRDWNECDYS  (88-98) | 2.097 | | | 11 |
|  | | | | | | | |
| 16 | T0597 | 3nie | MFEDLID  (64-70) | | 0.968 | | 7 |
|  | | | | | | | |
| 17 | T0599 | 3os6 | DYKTES  (17-22) | | 0.783 | | 6 |
|  | | | ASPTR  (27-31) | | 0.519 | | 5 |
|  |  |  | KHHEIE  (43-48) | | 0.914 | | 6 |
|  |  |  | HNKHGYT  (169-175) | | 0.975 | | 7 |
|  |  |  | RHGMQ  (202-206) | | 0.577 | | 5 |
|  | | | | | | | |
| 18 | T0601 | 3qtd | YAGQRK  (70-75) | | 0.98 | | 6 |
|  | | | STSATGEA  (79-86) | | 0.907 | | 8 |
|  |  |  | DKRVT  (151-155) | | 0.433 | | 5 |
|  |  |  | IAEGEGQM  (193-200) | | 1.054 | | 8 |
|  |  |  | VNRRGEALASA  (208-218) | | 1.186 | | 11 |
|  |  |  | SFLEGALGQRLFPEWLS  (273-289) | | 2.006 | | 17 |
|  |  |  | HLVGALGSASFDSDGLATYAK  (295-315) | | 5.633 | | 21 |
|  |  |  | FVSHGDEDQ  (351-359) | | 0.989 | | 9 |
|  |  |  | LMGQGVNLVTGDY  (375-387) | | 2.14 | | 13 |
|  | | | | | | | |
| 19 | T0603 | 3nkd | EAGVRVY  (80-86) | | | 1.575 | 7 |
|  | | | YASGQPGGARS  (86-96) | | | 2.511 | 11 |
|  |  |  | LYGVTEAAILAAGYAPAIGFVHTGKPL  (187-213) | | | 5.598 | 27 |
|  |  |  | KAFEIARRNPGEP  (231-243) | | | 2.568 | 13 |
|  | | | | | | | |
| 20 | T0609 | 3os7 | AAGGYE  (23-28) | | | 0.996 | 6 |
|  | | | IPDVGGNV  (32-38) | | | 1.075 | 7 |
|  |  |  | NYIHGFI  (105-111) | | | 1.385 | 7 |
|  |  |  | LSSKGLK  (158-164) | | | 1.139 | 7 |
|  |  |  | DMGDK  (290-294) | | | 0.813 | 5 |
|  | | | | | | | |
| 21 | T0613 | 3obi | PDRAGIV  (13-19) | | | 0.649 | 7 |
|  | | | FNAAAKVIP  (53-61) | | | 1.506 | 9 |
|  |  |  | VSQSDH  (95-100) | | | 0.457 | 6 |
|  |  |  | HFPVNKDTR  (142-150) | | | 1.223 | 9 |
|  | | | | | | | |
| 22 | T0615 | 3nqw | QYAAFKHRQQRRKDPQETPY  (14-33) | | | 5.086 | 20 |
|  | | | CITDE  (49-53) | | | 0.752 | 5 |
|  |  |  | LQVNTPTGWT  (131-140) | | | 1.817 | 10 |
|  |  |  | LRGTNA  (158-163) | | | 0.959 | 6 |
|  | | | | | | | |
| 23 | T0620 | 3nr8 | LKELTDLDY  (489-497) | | | 1.026 | 9 |
|  | | | SLWNIK  (504-509) | | | 0.565 | 5 |
|  |  |  | MFNGTS  (549-554) | | | 0.898 | 6 |
|  |  |  | CTDDIVTSDHS  (709-719) | | | 1.049 | 11 |
|  | | | | | | | |
| 24 | T0625 | 3oru | NRDGSQIGDF  (58-67) | | | 1.855 | 10 |
|  | | | AFVEGDCGEYLSME  (69-82) | | | 2.822 | 14 |
|  |  |  | DSSPGVHDTLVASCDVH  (113-129) | | | 5.905 | 17 |
|  |  |  | QLGHEGYHDNC  (133-143) | | | 2.303 | 11 |
|  | | | | | | | |
| 25 | T0626 | 3o1l | PDRVGIV  (11-17) | | | 0.911 | 7 |
|  | | | | | | | |
| 26 | T0632 | 3nwz | QLPDGQS  (101-107) | | | 0.8 | 7 |
|  | | | TDQGET  (148-153) | | | 0.353 | 6 |
|  | | | | | | | |
| 27 | T0638 | 3nxh | SENVETNIRITEGLALQ  (263-279) | | | 3.659 | 17 |
|  | | | | | | | |
| 28 | T0640 | 3nyw | SNKHVQEP  (52-59) | | | 1.423 | 8 |
|  | | | MFMDGSLSEPV  (95-105) | | | 2.387 | 11 |
|  |  |  | KAGTPFK  (196-202) | | | 1.488 | 7 |
|  | | | | | | | |
| 29 | T0641 | 3nyi | VEQGFPV  (78-84) | | | 1.162 | 7 |
|  | | | DYPDAN  (110-115) | | | 0.448 | 6 |

**Figure S1**

**(a)**


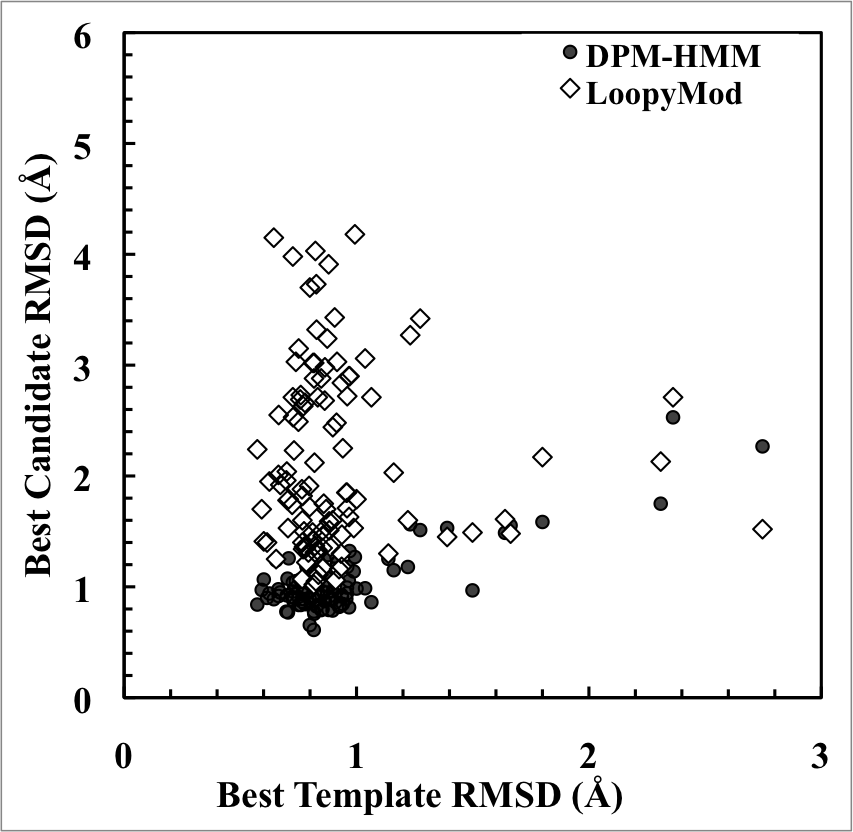


**(b)**

**
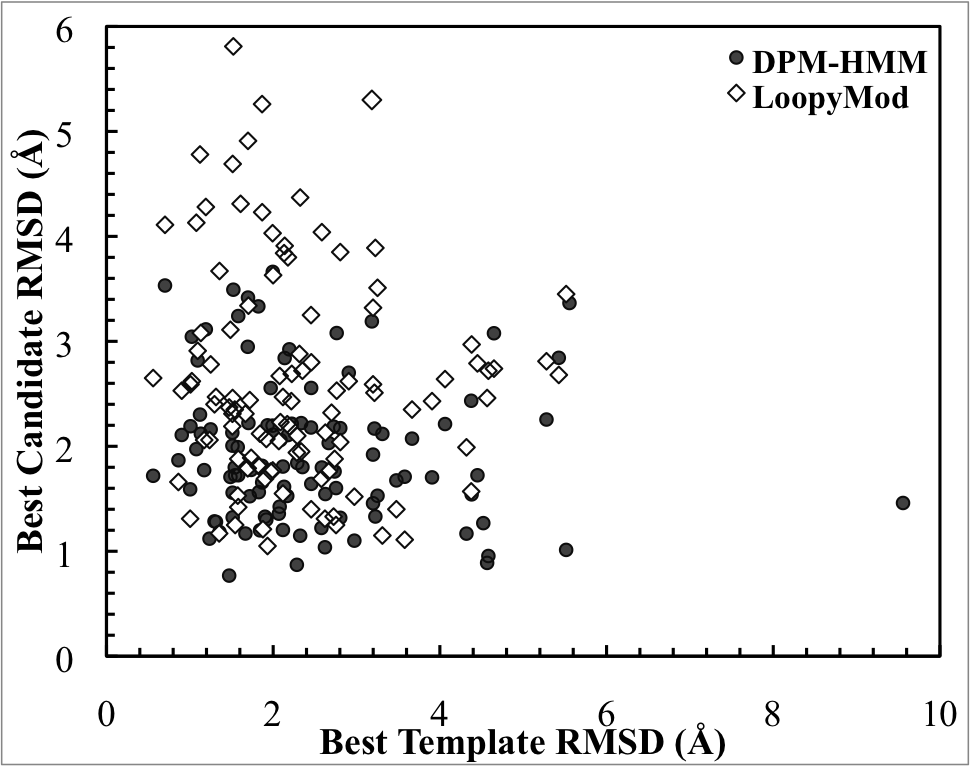
**
